# Outcomes of real‐world initiation of long‐acting injectable cabotegravir and rilpivirine (LA‐I CAB + RPV) in individuals with viraemia: A systematic review of baseline characteristics, virological failure outcomes and discontinuations

**DOI:** 10.1111/hiv.70113

**Published:** 2025-09-13

**Authors:** Alexa Elias, Chloé Pasin, Melanie Smuk, Amy Paterson, Chloe M. Orkin

**Affiliations:** ^1^ SHARE Collaborative, Blizard Institute Queen Mary University of London London UK; ^2^ Pandemic Sciences Institute University of Oxford Oxford UK; ^3^ Barts Health NHS Trust London UK

**Keywords:** cabotegravir + rilpivirine, real‐world, resistance, viraemic, virological failure

## Abstract

**Background:**

When using long‐acting injectable cabotegravir and rilpivirine (CAB + RPV) in individuals with viraemia, beyond small cohorts, little is known about baseline characteristics, virological failure outcomes, resistance emergence, re‐suppression and discontinuation.

**Methods:**

We identified evidence from PubMed, EMBASE, Cochrane and conference abstract databases through 17 March 2025 to synthesize data from observational cohort studies (OCS) that reported on virological failure (VF) events in individuals with viraemia at initiation of LA‐I CAB + RPV. We extracted data on baseline clinical and socio‐demographic characteristics, VF, resistance‐associated mutations (RAMs) at VF, post‐VF regimen choice, re‐suppression and discontinuation.

**Results:**

Across 14 cohorts including 561 individuals, there were 36 VF events (OCS‐level VF rate 0% (*n*/*N* = 0/12, 0/12 and 0/10) to 25% (*n*/*N* = 3/12)). VF definitions varied. 6/14 OCS (*n* = 436) reported on baseline CD4 count, 13/14 (*n* = 543) on baseline viral load and 7/14 (*n* = 459) on socio‐demographic characteristics. Among the 14 VF events with genotypic information available at VF, NNRTI RAMs were identified in 13/14 individuals, INI RAMs in 9/14 and dual‐class resistance in 8/14 individuals. Post‐VF regimens were reported for 16/36 individuals with VF and included lenacapavir (LEN)‐based regimens, protease inhibitor (PI)‐based regimens or LA‐I CAB + RPV continuation. Re‐suppression outcomes were described in 10 VF events: re‐suppression occurred in 5/10.

**Conclusions:**

In OCS, the follow‐up duration was short and VF definitions were highly variable, with few cohorts reporting VF outcomes in people with baseline VL >10 000 c/mL. VF was frequently accompanied by resistance. Post‐VF regimens varied, and their success was unclear due to the small sample size.

## INTRODUCTION

For most people living with HIV, oral ART has transformed life expectancy, provided they are able and willing to take it every day. However, viraemia while on ART together with disengagement from care still costs lives and is a pressing challenge for people living with HIV. Longer‐acting modalities are more discreet and offer freedom from daily pill‐taking. The first long‐acting intramuscular injectable therapy for people living with HIV‐1, cabotegravir and rilpivirine (LA‐I CAB + RPV), is recommended to maintain viral suppression in international guidelines for people who are already virally suppressed [1, 2, 3]. Exclusions include Hepatitis B antigenaemia resistance to and/or prior virological failure on non‐nucleoside reverse transcriptase inhibitors (NNRTIs) or integrase strand transfer inhibitors (INIs) [4, 5].

Clear benefits to adherence and persistence on LA‐I CAB + RPV versus oral therapy have been demonstrated in people who experience psychosocial challenges on oral therapy, including adolescent populations [6, 7, 8, 9]. Evidence from prospective observational clinical cohorts also indicates that long‐acting injectable (LA‐I) CAB + RPV is beneficial for people experiencing intra‐ and interpersonal stigma [8, 10, 11].

From an effectiveness standpoint, observational cohort studies in around 14 000 virally suppressed individuals showed that the risk of virological failure (VF) is low (ranging between 0% and 2.41%) and comparable to clinical studies [1]. However, emergent resistance at VF to either (or both) NNRTIs and INIs may limit future treatment options with INI‐based regimens [6, 12, 13, 14, 15, 16, 17, 18]. Previous clinical studies have identified risk factors for VF, including the presence of archived rilpivirine resistance‐associated mutations, HIV‐1 subtype A6 and body mass index greater than 30 kg/m^2^ [19]. In the presence of 2 or more risk factors, the risk of VF rises to 19%, which needs to be carefully considered to guide appropriate use [19]. It is noteworthy that BMI alone is not associated with VF [20, 21].

In individuals with viraemia at the time of LA‐I CAB + RPV initiation, evidence on the efficacy of LA‐I CAB + RPV is scant. To date, there have been no randomized studies evaluating the regimen in individuals with viraemia at initiation of LA‐I CAB + RPV. A recent interim analysis of a phase III, prospective, randomized, open‐label trial study enrolling people who were not virally suppressed at baseline but were suppressed to <200 c/mL for at least 4 weeks at the time of switch to LA‐I CAB + RPV (LATITUDE, ACTG A5359) showed that LA‐I CAB + RPV was superior to oral ART regarding regimen failure [22]. This led to a drug safety monitoring board decision to suspend the randomized phase of the trial and offer LA‐I CAB + RPV to all participants. Notably, to achieve viral suppression at the time of switch to LA‐I CAB + RPV, the participants received conditional economic incentives and a three‐drug oral ART regimen. Additionally, the IMPALA study for people with viraemia with poor adherence and/or virological control involved a 3‐month non‐incentivized suppression period on oral ART. It showed non‐inferiority to oral ART at 48 weeks [7]. There were 5 VF events; dual‐class resistance occurred in 4/5 individuals (one was not known or ongoing), all of whom re‐suppressed; 4/5 reached viral suppression on dolutegravir‐based therapy.

While initial treatment guidelines at the time of licensing did not recommend LA‐I CAB + RPV use in people with viraemia, DHHS and IAS‐USA guidelines have since been amended to support use in a narrow range of clinical circumstances [23, 24]. This change followed early positive results from clinical practice in pioneering clinics such as Ward 86 [25, 26, 27] where LA‐I CAB + RPV was prescribed monthly for the first 3 months according to strict approval and monitoring protocols. The guidelines in people with viraemia now suggest decision‐making on a case‐by‐case basis in individuals at high risk of HIV disease progression who encounter persistent difficulties taking oral ART and in whom there is no known or suspected viral resistance to either component drug [23, 24, 28]. Guidelines emphasize that this should be implemented together with intensive monitoring, adherence support and substance misuse support where appropriate, to maximize the chance of virologic control [23, 24, 28].

Results from other small real‐world observational cohort studies seem similarly encouraging in the context of careful inclusion protocols around prior VF and resistance. A recent early meta‐analysis evaluated LA‐I CAB + RPV effectiveness in people with viraemia. In this meta‐analysis, the virologic success rate was 87% (95% confidence interval: 79%–95%) and the adherence rates were high (mostly above 90%) [29] However, this meta‐analysis included only 5 manuscripts and 3 oral abstracts (*n* = 244 individuals) with a short follow‐up duration, and it did not assess other variables—such as CD4+ cell count or viral load at baseline, nor the development of resistance‐associated mutations (RAMs) to RPV or CAB, suppression regimens or re‐suppression rates.

We present a detailed evidence synthesis based on the results of a large, clinically focused systematic review focused on real‐world observational cohort studies (OCS) and case series in viraemic individuals. We describe baseline characteristics and outcomes—including VF, RAMs, discontinuations, treatment regimens post VF and re‐suppression outcomes where known.

## METHODS

The OUTCOMES Study is a 2‐year evidence synthesis project based on an ongoing systematic literature review. Its objective is to summarize evidence from clinical practice as reported in OCS from both published and grey literature regarding virological outcomes in individuals with HIV who transition to LA‐I CAB + RPV in various clinical contexts (namely virally suppressed and non‐suppressed populations). Guidance from the Cochrane handbook [30] and Preferred Reporting Items for Systematic Reviews and Meta‐Analysis (PRISMA) guidelines [31] highlight the need for studies to be sufficiently comparable to justify statistical pooling in a meta‐analysis. However, the included studies were highly heterogeneous in terms of reported data, length of follow‐up and outcome (VF) definition. Given these limitations, we used a narrative synthesis approach and followed the PRISMA guidelines. The protocol for the OUTCOMES Study was approved on June 10, 2024 (https://doi.org/10.17605/OSF.IO/V68UN).

This manuscript pertains to Phase 2 of the OUTCOMES Study, which focuses on virological outcomes in individuals with viraemia at the time of switch to LA‐I CAB + RPV. The methods of the OUTCOMES study have been previously published in a manuscript which focussed on virological outcomes in virally suppressed individuals at the time of switch [1]. Search strategy, study screening and inclusion criteria, data extraction and quality assessment methods using a modified Down's and Black checklist are published [1] and are described in the Supplemental methods. As per the protocol, we designated OCS studies as n>10. Data from OCS with fewer than 10 individuals with viraemia and case series were summarized in a separate table.

### Outcomes and data analysis

We summarized the total number of individuals who were at risk of VF and/or resistance in each OCS. We summarized the number of OCS reporting on socio‐demographic and clinical characteristics at baseline (age, gender, number of racially minoritized participants, unstable housing/unhoused, substance misuse, mental health problems, number of prior regimes, time from HIV diagnosis, baseline CD4 counts). This is available in Table 2.

We reported VF events by individual in each study. We recorded a VF definition for each cohort where described. Where a VF definition was not explicitly defined, we have used another marker of VF (such as resistance or VL exceeding the specified VL cut‐off, such as VL 200 c/mL) mentioned in the abstract and recorded this as the VF definition. We have labelled the definition as not reported if the study stated the presence or absence of VF but did not define it.

We descriptively summarized the number of VF events observed, and, where available, genotypic data on RAM presence at VF and at baseline (and/or historical RAMs), post‐VF regimens and re‐suppressions post VF. We considered only RAMs that are reported as NNRTI or INI RAMs by the Stanford HIV Drug Resistance Database. We categorized follow‐up durations into 6‐month periods. We classified post‐VF ART regimens by anchor agent as: oral integrase inhibitor (INI), protease inhibitor (PI), non‐NNRTI, CAB ± RPV regimens where injectable Lenacapavir (LEN) was added and continued LA‐I CAB + RPV. We have reported the number of participants with re‐suppression, where known. This is available in Table 3.

We reported the number of OCS that reported on discontinuations and the number of discontinuations of LA‐I CAB + RPV within those cohorts.

We also separately summarized baseline socio‐demographic and clinical characteristics, VF, resistance and re‐suppression for OCS including <10 individuals with viraemia and in case series. This is available in Tables S10 and S11.

*Role of the funding source*: No writing support was provided. Inizio Medical was funded to perform the literature search and primary screening as outlined in the protocol.

## RESULTS

### Literature search

**FIGURE 1:**
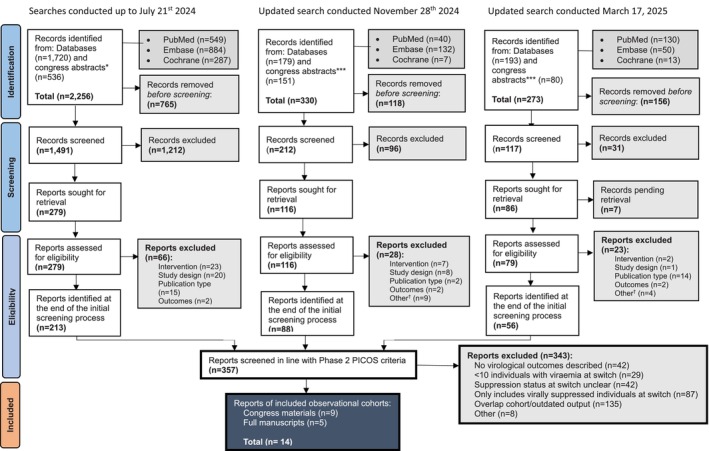
PRISMA flow diagram of search results.

The literature search for the OUTCOMES Study initially identified 2256 records (Figure 1). After de‐duplication, titles and abstracts from 1491 records were screened. The remaining 279 were screened using full texts. Eligible records from two separate updated searches (*n* = 88, *n* = 56, respectively) were then added to the remaining initial 213 records. A combined total of 357 records identified from all three searches were then screened again using a focused set of inclusion criteria, resulting in 14 included OCS (9 congress materials, 5 full manuscripts) evaluating the use of LA‐I CAB + RPV in individuals with viraemia at the time of switch [32, 33, 34, 35, 36, 37, 38, 39, 40, 41, 42, 43, 44, 45].

The 14 OCS included in our review described outcomes in 561 individuals with viraemia, with OCS size ranging from 10 to 176 (Table 2). Of these, 4/14 included only individuals with viraemia (*n* = 224), and 10/14 reported data from both virally suppressed individuals and individuals with viraemia (*n* = 337). All studies were based in high‐income countries, with 10/14 conducted in the USA, 3/14 in Europe, and one multi‐country study (USA, Europe and South Korea). Half of the observational cohorts were multi‐site cohorts.

13/14 OCS reported on the follow‐up time, which was 1 year or less in 11/14 OCS (*n* = 375 individuals). 10/14 OCS (including 487 individuals) reported on LA‐I CAB + RPV discontinuation. In 9/14 OCS, the injection schedule was specified at the time of switch, with 4 prescribing LA‐I CAB + RPV 2‐monthly, 1 prescribing LA‐I CAB + RPV 1‐monthly and 4 prescribing LA‐I CAB + RPV using an induction maintenance strategy (i.e., monthly at first and then 2‐monthly).

### Baseline characteristics

**FIGURE 2:**
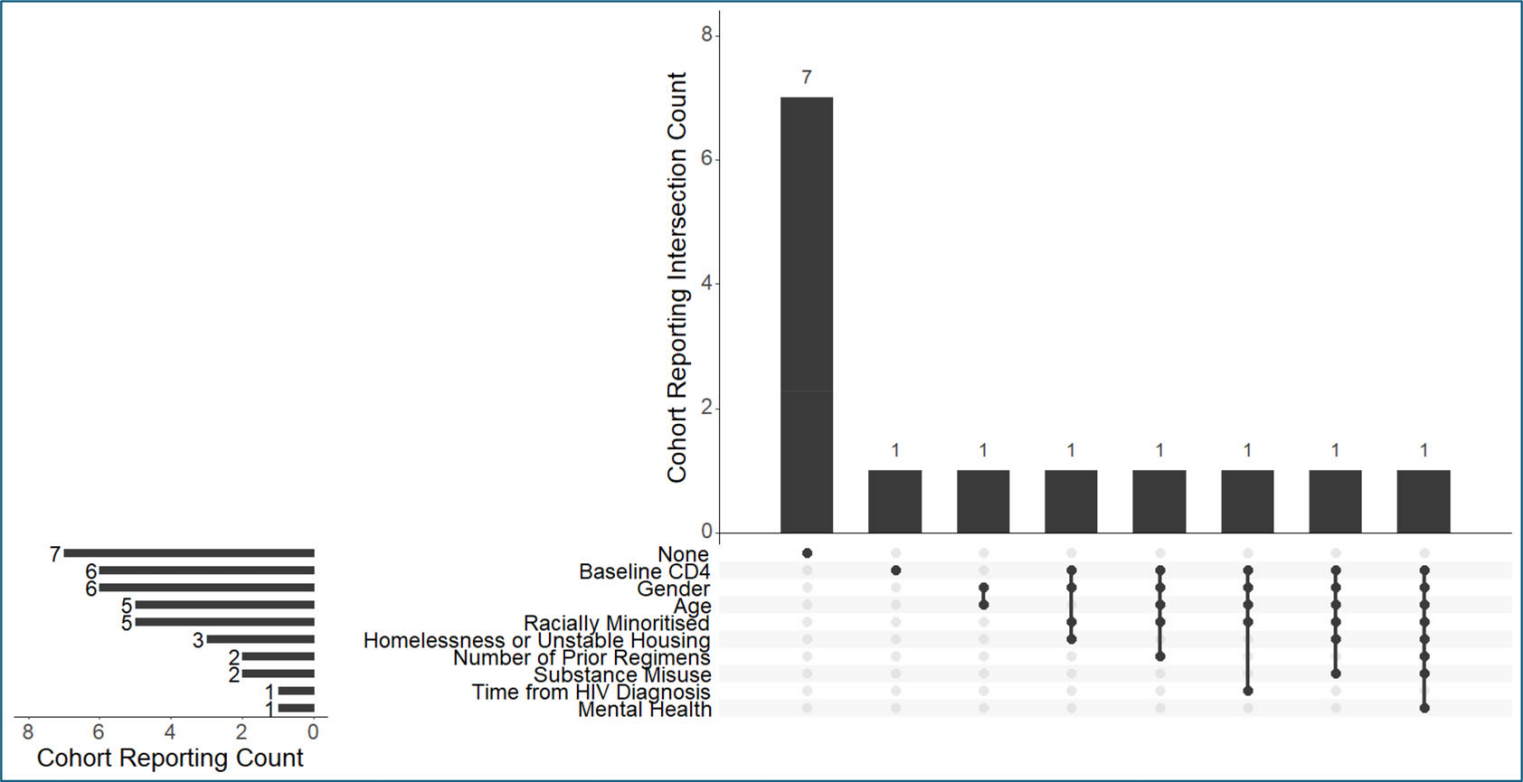
Cohort‐level reporting on baseline characteristics for individuals with viraemia initiating LAI‐CAB + RPV. We indicate reporting only of discrete data describing individuals with viraemia initiating LAI‐CAB + RPV alone. We exclude reporting of data inclusive of individuals who were virally suppressed or on LAI‐CAB + RPV alongside additional ART.

Baseline characteristics were heterogeneously reported (Figure 2, Table 2): half of the OCS (7/14) did not report on any participant characteristics. 6/14 OCS provided information on gender, of which 4/6 further provided information on female participants (reported by OCS as either female gender or sex). In those, 22% (87/400) of participants were assigned female at birth. In addition, 3/6 OCS specified whether transgender women (TGW) were included. Within these cohorts, TGW represented 10% (*n* = 14/151) of the cohort. 5/14 OCS reported on ethnicity at baseline. In these cohorts, 68% (*n* = 270/400) individuals were from racially minoritized backgrounds. 5/14 OCS reported data on age, out of which 4 recruited participants 50 years old and older, and one was focused on youth living with HIV. Only three OCS reported on whether individuals were unhoused or experiencing unstable housing, two OCS reported on substance misuse and one OCS (*n* = 12) reported on mental health. Baseline CD4 counts were reported in 6 OCS, with 3/6 (*n* = 273) reporting the median/mean CD4 counts in the population, and 3/6 (*n* = 163) reporting on the number of participants with CD4 counts below and above 200 cells/μL. In these studies, the median/mean CD4 counts varied between 233 and 770 cells/μL, and the proportion of participants with CD4 counts below 200 cells/μL varied between 63% and 79%. Concerning baseline VL at the time of switch, 6 OCS specified that individuals with HIV‐RNA VL >10 000 c/mL were included, 7 OCS included individuals with VL 50 to 200 and 2 included individuals described as VL >50 (Tables 1, 2 and 4).

**TABLE 1:** Baseline CD4 count and viral load at LAI‐CAB + RPV initiation.

Study reference, value	Data provided by cohort	Total individuals with viraemia with data available, *n*
Baseline CD4, cells/μL		
OPERA^1^, median [IQR]	579 [350–759]	229^a^
Ward 86^2^ (*n*)	<200 (88); ≥200 (30)^b^	118^b^
Trio^3^, median [IQR]	770 [619–1042]	32^c^
Compassionate Use^4^ (*n*)	<200 (22); ≥200 (6)	28
Gerber et al. (2025)^5^ (*n*)	<200 (11); ≥200 (6)	17
Brock et al. (2024)^6^, mean [range]	233 [131–475]	12
Baseline viral load, c/mL^d^		
OPERA^1^ (*n*)	VL 50–199 (136); VL ≥200 (93)	229^a^
Ward 86^2^, (*n*)	VL ≥50 (150)^e^	150
Hill et al. (2025)^7^ (*n*)	VL ≥50 (35)	35
Trio^3^ (*n*)	VL 50–199 (18); VL ≥200 (14)	32^c^
Fessler et al. (2024)^8^, median [range]	VL 160 [60–257 000]	31
Compassionate use^4^, median [range]	VL 60 300 [86–>10 000 000]	28
Dutch ATHENA^9^ (*n*)	VL 50–199 (13); VL >200 (5)	18
BEYOND^10^ (*n*)	VL ≥50 (18)	18
Gerber et al. (2025)^5^, median	VL 21 045	17
Brock et al. (2024)^6^, mean [range]	VL 152657 [2410‐566 000]	12
Hessamfar et al. (2024)^11^ (*n*)	VL 51–200 (5) VL >200 (7)	12
Rousseau et al. (2024)^12^ (*n*)	VL 50–10 000 (5); VL >10 000 (7)	12
O'Connor et al. (2025)^13^ (*n*)	VL ≥200 (10)	10

*Note*: *References*: (1) Hsu et al. IDWeek 2023. Oral 1028 (2) Christopoulos et al. CROI 2025. Poster 0683 (3) Elion et al. 2023 ID Week P.1592 (4) D'Amico et al. HIV Med 2023;24:202–11 (5) Gerber et al. CROI 2025. Poster 682 (6) Brock et al. Clin Infect Dis 2024;78:122–4 (7) Hill et al. J Acquir Immune Defic Syndr 2025;98:185–192 (8) Fessler et al. CROI 2024. Poster 1235 (9) Jongen et al. Lancet HIV 2025; 12: e40–50 (10) Schneider et al. AIDS 2024. Poster THPEB099 (11) Hessamfar et al. HIV Glasgow 2024. Poster P078 (12) Rousseau et al. J Pediatric Infect Dis Soc 2024;13:285–87 (13) O'Connor et al. CROI 2025. Poster 691.

Abbreviations: IQR, interquartile range; VL, viral load.

^a^ Only 176 had follow‐up data.

^b^ Data on CD4 was extracted from Gistand et al., CROI 2025 Poster 0689 and describes individuals with VL > 30 at switch.

^c^ Only 24 had follow‐up data.

^d^ 1/14 RWCs (Dawiec et al., HIV Glasgow 2025. Poster 097) did not provide data on the baseline VL of those indicated as viraemic at switch and is therefore not listed.

^e^ Data provided for a subgroup that discontinued LAI‐CAB + RPV indicates that at least 9 individuals with viraemia had VL > 10 000 at switch.

**TABLE 2:** Study characteristics and baseline characteristics of individuals with viraemia initiating LA‐I CAB + RPV in observational cohort studies.

Study characteristics							Baseline characteristics										
Study	Total people with viraemia at switch to CAB + RPV, *n*	Type of cohort	Study country	Multi‐site	Schedule of injections	Follow‐up time	Total people with available data on baseline characteristics	Include people with VL >10 000	CD4 counts (cell/μL)	Reports on gender	Reports on age	Reports on racially minoritized people	Reports on substance misuse	Reports on mental health	Reports on homelessness or unstable housing	Reports on time from HIV diagnosis	Reports on number of prior regimens
1. Schneider et al. (2024) (BEYOND)	18	Mixed	USA	Yes	NR	Through 12	0	NR	NR	No	No	No	No	No	No	No	No
2. Hessamfar et al. (2024)	12	Mixed	France	Yes	Q2M	Through 12	0	NR	NR	No	No	No	No	No	No	No	No
3. Dawiec et al. (2024)	18	Mixed	Poland	No	Q2M	Over 12	0	NR	NR	No	No	No	No	No	No	No	No
4. Jongen et al. (2025) (Dutch ATHENA)	18	Mixed	Netherlands	Yes	Q2M	Over 12	0	NR	NR	No	No	No	No	No	No	No	no
5. Fessler et al. (2024)	31	Mixed	USA	No	NR	Less than 6	31	Yes	NR	No	No	No	No	No	No	No	no
6. Christopoulos et al. (2025) (Ward 86)	150	Mixed	USA	No	Q1M and Q2M	NR	129^a^	Yes	30/118 CD4 ≥ 200; 88/118 CD4 < 200	Yes	Yes	Yes	Yes	No	Yes	No	No
7. Elion et al. (2023) (Trio)	24	Only people with viraemia	USA	Yes	Q1M and Q2M	Less than 6	32^b^	NR	1770 (619–1042) (median *n* = 32)	Yes	Yes	Yes	No	No	No	No	Yes
8. D'Amico et al. (2023) (Compassionate Use)	28	Mixed	UK, USA, Belgium, Canada, Netherlands, Switzerland, Portugal, Italy, South Korea, Spain	Yes	Q1M	Less than 6	28	Yes	22/28 CD4 > 200; 6/28 CD4 < 200	No	No	No	No	No	No	No	No
9. Hsu et al. (2023) (OPERA)	176	Only people with viraemia	USA	Yes	Q1M and Q2M	Through 6	229^c^	NR	2579 (350–759) (median *n* = 229)	Yes	Yes	Yes	No	No	No	Yes	No
10. Brock et al. (2024)	12	Only people with viraemia	USA	No	Q1M and Q2M	Less than 6	12	Yes	233 cells/μL (mean) (*n* = 12)	Yes	Yes	Yes	Yes	Yes	Yes	No	Yes
11. Hill et al. (2025)	35	Mixed	USA	No	NR	Through 6	35	Yes	NR	No	No	No	No	No	No	No	No
12. Rousseau et al. (2024)	12	Only people with viraemia	USA	No	Q2M	Through 6	12	Yes	NR	Yes	Yes	No	No	No	No	No	No
13. O'Connor et al. (2025)	10	Mixed	USA	No	NR	Less than 6	0	NR	NR	No	No	No	No	No	No	No	No
14. Gerber et al. (2025)	17	Mixed	USA	Yes	NR	Less than 6	17	Yes	63% of viraemic individuals had CD4 < 200 CP/ML	Yes	No	Yes	No	No	Yes	No	No

*Note*: *References*: (1) Schneider et al. AIDS 2024. Poster THPEB099 (2) Hessamfar et al. V Glasgow 2024. Poster P078 (3) Dawiec et al. HIV Glasgow 2025. Poster 097 (4) Jongen et al. Lancet HIV 2025; 12: e40–50 (5) Fessler et al. CROI 2024. Poster 1235 (6) Christopoulos et al. CROI 2025. Poster 0683 (7) Elion et al. 2023 ID Week P.1592 (8) D'Amico et al. HIV Med 2023;24:202–11 (9) Hsu et al. IDWeek 2023. Oral 1028 (10) Brock et al. Clin Infect D 2024;78:122–4 (11) Hill et al. J Acquir Immune Defic Syndr 2025;98:185–192 (12) Rousseau et al. J Pediatric Infect Dis Soc 2024;13:285–87 (13) O'Connor et al. CROI 2025. Poster 691 (14) Gerber et al., CROI 2025. Poster 682. Grey shade indicates when the data was not available (i.e., *n* = 0 people with characteristic available, or “NR”).

Abbreviations: NR, not reported; Q1M, monthly; Q2M, 2‐monthly.

^a^ Data on CD4 was extracted from Gistand et al., CROI 2025 Poster 0689 and describes individuals with VL > 30 at switch.

^b^ Only 24 had follow‐up data.

^c^ Only 176 had follow‐up data.

### Virological failure definition

Virological failure was mostly defined as a single elevated HIV‐RNA viral load (*n* = 10/14 OCS) above a 50 c/mL or 200 c/mL threshold (Tables 3 and 4). One study defined VF as an elevated VL with a subsequent confirmatory elevated HIV‐RNA VL, two studies used complex definitions and one study did not report on the definition they used for VF. Given that only 2 studies (Jongen et al. and Hill et al) followed the recent consensus definition of VF [46], we were not able to conduct any sensitivity analysis on those.

**TABLE 3:** Summary table of follow‐up durations and key virological definitions.

Total OCS included, *N*	14
Total individuals with viraemia at risk of VF^a^	561
OCS follow‐up duration, *n* (*n*/*N*%)	
Through 6 months	6 (42.9%)
Less than 6 months	3 (21.4%)
Through 12 months	2 (14.3%)
Over 12 months	2 (14.3%)
Not reported	1 (7.1%)
VF definitions used, *n* (*n*/*N*%)	
Single elevated VL	10 (71.4%)
Confirmatory VL	1 (7.1%)
Other complex definition	2 (14.3%)
Not reported	1 (7.1%)
OCS that describe presence/absence of VF, *n*	14
OCS with VF events	11
OCS without VF events	3
Individuals at risk of VF^a^, *n*	561
VF events, *n*	36
OCS which focus on reporting both VF and presence/absence of RAMs at VF, *n*	4
Individuals at risk of RAMs^a^, *n*	214
VF events with corresponding data on presence/absence of RAMs, *n*	14
VF events with RAMs identified	14
VF events where no RAMs were observed	0
RAMS by drug class^b^, *n*	
NNRTI RAMs observed	13
INI RAMs observed	9
NNRTI and INI RAMs observed	8
OCS with a focus on post‐VF regimen data, *n*	4
Individuals at risk of VF^a^, *n*	372
VF events with post‐VF regimen data provided, *n*	16
Regimen type	
PI, *n* (*n*/*N*%)	8 (50%)
LA‐I CAB + RPV	3 (19%)
CAB/RPV + LEN	1 (6%)
CAB/RPV + PI	1 (6%)
PI + LEN	1 (6%)
INI + LEN	2 (13%)
OCS with a focus on re‐suppression outcomes post VF, *n*	3
Individuals at risk of VF^a^, *n*	196
VF events with known re‐suppression outcome, *N*	10
Re‐suppressed, *n* (*n*/*N*%)	5 (50%)
Not re‐suppressed	5 (50%)
OCS reporting on discontinuations, *n*	10
Individuals at risk of discontinuation, *n*	487
Discontinuations, *n*	35

Abbreviations: INI, major integrase inhibitor; LA‐I CAB + RPV, long‐acting injectable cabotegravir and rilpivirine; LEN, lenacapavir; NNRTI, non‐nucleoside reverse transcriptase inhibitor; OCS, observational cohort studies; PI, protease inhibitor; RAMs, resistance‐associated mutations; VF, virological failure; VL, viral load.

^a^ Individuals included in the VF analysis.

^b^ Categories are not mutually exclusive.

**TABLE 4:** Data on VF, resistance, post‐VF regimens and re‐suppression in individuals with viraemia initiating LA‐I CAB + RPV in observational cohort studies.

Study characteristics			VF				RAM data				Post‐VF data				Discontinuations	
Study	Total people with viraemia at switch to CAB + RPV, *n*	VL at switch to CAB + RPV (c/mL)	VF definition	VF, *n* (*n*/*N*%)	VL at VF (c/mL)	VL at switch to CAB + RPV (c/mL)	Genotype available at VF, *n*	VF with NNRTI RAM, *n* (*n*/*N*%)	VF with INI RAM, *n* (*n*/*N*%)	Genotypic information available on baseline and/or historic RAM for those with genotypic information at VF, *n*	VF events with post‐VF regimen data	Post‐VF regimen type	VF events with known re‐suppression outcome	Re‐suppressed	Reports on discontinuations	Discontinuations, *n* (%)
1. Schneider et al. (2024) (BEYOND)	18	VL ≥ 50 (no numerical value available)	VL ≥ 50 c/mL^a^	1 (5.6)	VL ≥ 50 (no numerical value available)	VL ≥ 50 (no numerical value available)	NR	NR	NR	NR	NR	NR	NR	NR	No	NA
2. Hessamfar et al. (2024)	12	*n* = 5 with VL 51–200 *n* = 7 with VL ≥ 200	VL ≥ 50 c/mL at 6 months	3 (25.0)	VL ≥ 50 (no numerical value available)	VL ≥ 50 (no numerical value available)	NR	NR	NR	NR	NR	NR	NR	NR	No	NA
3. Dawiec et al. (2024)	18	NR	NR	2 (11.1)	VL >1000 (no numerical value available)	NR	1/2	1 (5.6) mutations NR^a^	1 (5.6) mutations NR^a^	1/1 mutations NR^a^	NR	NR	NR	NR	Yes	0
4. Jongen et al. (2025) (Dutch ATHENA)	18	*n* = 13 with VL 50–199 *n* = 5 with VL ≥ 200	VF: VL ≥ 1000 c/mL + ART change or RAMs CVF: >2 VL > 200 c/mL or 1 VL >200 c/mL with CAB or RPV RAMs	1 (5.6)	VL = 200	VL = 100	1/1	0 (0.0)	1 (5.6) N155H	1/1 None	1	1: PI	1	1: no	Yes	1 (5.6)
5. Fessler et al. (2024)	31	Median VL 160 Range VL 60–257 000	VL > 50 c/mL	2 (6.5)	VL = 60 VL = 60	VL ≥ 50 (no numerical value available)	NR	NR	NR	NR	NR	NR	NR	NR	Yes	0
6. Christopoulos et al. (2025) (Ward 86)^b^	150	VL ≥ 30 (no numerical value available)	VL ≥ 200 c/mL	6 (4.0)	VL = 4400 VL = 29 000 VL = 4500 VL = 1300 VL = 137 000 VL = 8600	VL = 137 000 VL = 215 000 VL = 363 800 VL = 700 VL = 309 VL = 540 000	6/6	6 (4.0) E138K L100I, Y181I K101E K101K/E, K103N, Y181Y/C M230L M230L, K101Q	4 (2.7) R263K None Q148R E138E/K, Q148K None E138E/D/K/N, G140G/S, S147S/G, Q148K	5/6 T97A (INI) V179I, N348I (minor NNRTI) n/a K103N None V90I	6	INI + LEN INI + LEN CAB/RPV + LEN PI PI PI + LEN	4	Yes Yes Yes Yes NA NA	Yes	22 (14.7)
7. Elion et al. (2023) (Trio)	24	*n* = 14 with VL 50–199 *n* = 10 with VL ≥ 200	VL ≥ 200 c/mL	3 (12.5)	VL = 205 VL = 1148 VL = 3715	VL 50–199 (no numerical value) VL = 2138 VL = 616	NR	NR	NR	NR	NR	NR	NR	NR	Yes	1 (3.1)
8. D'Amico et al. (2023) (Compassionate Use)	28	Median VL 60 300 range VL 86 to >10 000 000 *n* = 11 with VL >100 000	VL ≥ 50 c/mL	6 (21.4)	NR VL = 799 VL = 37 594 VL = 7190 VL = 186 972 VL = 66 000	VL = 61 600 VL = 32 000 VL = 116 311 VL = 77 578 VL = 205 000 VL = 1 639 794	6/6	6 (21.4) E138E/K E138G, M230L E138G, Y181I K101E K101E, Y181Y/C Y181C	3 (10.7) None Q148R, N155H M50M/I, E157E/Q E138E/K, Q148R None None	6/6 E138G K238K/R, E138G V179I None K103N K103N	6	CAB/RPV + PI PI PI PI PI PI	5	No No NA No No Yes	Yes	6 (21.4)
9. Hsu et al. (2023) (OPERA)	176	*n* = 107 with VL 50–199 *n* = 69 with VL ≥ 200	2 VL ≥ 200 c/mL or 1 VL ≥ 200 + discontinuation (ART change or 2 missed injections)	7 (4.0)	VL ≥ 200 (no numerical value available)	VL ≥ 50 (no numerical value available)	NR	NR	NR	NR	3	CAB + RPV *n* = 3	NR	NR	Yes	5 (2.8)
10. Brock et al. (2024)	12	Mean VL 152 657 range VL 2410–566 000	VL ≥ 200 c/mL	0	NA	NA	NR	NR	NR	NR	NR	NR	NR	NR	Yes	0
11. Hill et al. (2025)	35	VL ≥ 50 (no numerical value available)	2 VL > 200 c/mL	1 (2.9)	No numerical value available	No numerical value available	NR	NR	NR	NR	NR	NR	NR	NR	No	NA
12. Rousseau et al. (2024)	12	*n* = 5 with VL 50–9999 *n* = 7 with VL ≥ 10 000	VL ≥ 50 c/mL	0	NA	NA	NR	NR	NR	NR	NR	NR	NR	NR	Yes	0
13. O'Connor et al. (2025)	10	VL ≥ 200 (no numerical value available)	VL ≥ 200 c/mL	0	NA	NA	NR	NR	NR	NR	NR	NR	NR	NR	Yes	0
14. Gerber et al. (2025)	17	Median VL 21 045 range VL 390–152 997	VL ≥ 200 c/mL	4 (23.5)	VL ≥ 200 (no numerical value available)	VL ≥ 200 (no numerical value available)	NR	NR	NR	NR	NR	NR	NR	NR	No	NA

*Note*: *References*: (1) Schneider et al. AIDS 2024. Poster THPEB099 (2) Hessamfar et al. V Glasgow 2024. Poster P078 (3) Dawiec et al. HIV Glasgow 2025. Poster 097 (4) Jongen et al. Lancet HIV 2025; 12: e40–50 (5) Fessler et al. CROI 2024. Poster 1235 (6) Christopoulos et al. CROI 2025. Poster 0683 (7) Elion et al. 2023 ID Week P.1592 (8) D'Amico et al. HIV Med 2023;24:202–11 (9) Hsu et al. IDWeek 2023. Oral 1028 (10) Brock et al. Clin Infect Dis 2024;78:122–4 (11) Hill et al. J Acquir Immune Defic Syndr 2025;98:185–192 (12) Rousseau et al. J Pediatric Infect Dis Soc 2024;13:285–87 (13) O'Connor et al. CROI 2025. Poster 691 (14) Gerber et al. CROI 2025. Poster 682. Grey shade indicates when the data was not available (i.e., *n* = 0 people with characteristic available, or “NR”).

Abbreviations: FU, follow‐up; INI, integrase inhibitor; LA‐I CAB + RPV, long‐acting cabotegravir and rilpivirine; NNRTI, non‐nucleoside reverse transcriptase inhibitor; NR, Not reported; PI, protease inhibitor; RAMs, resistance‐associated mutations; VF, viral failure; VL, viral load.

^a^ Although a VF definition with a confirmatory viral load was utilized for participants inconsistent with the label, we considered viral suppression as the definition of VF here because it was the only virological outcome reported discretely for participants with viraemia at baseline.

^b^ Data on baseline VL was extracted from Gistand et al., CROI 2025 Poster 0689.

### Duration of follow‐up

Almost all OCS (13/14) reported on the duration of follow‐up: 3/13 reported time on LA‐I CAB + RPV through 6 months, 6/13 less than 6 months, 2/13 through 12 months and 2/13 longer than 12 months (Tables 2 and 3).

### Virological failure events

Out of the 14 OCS, 36 VF events occurred across 11 studies (among 527 individuals). A summary of those is available in Table S9. The OCS size ranged from 10 to 176 individuals at risk (Tables 3 and 4). Across OCS, VF rate ranged from 0% (*n*/*N* = 0/12, 0/12 and 0/10) to 25% (*n*/*N* = 3/12). In the two OCS with more than 100 individuals, VF was 4% (*n*/*N* = 6/150 and 7/176). At the individual level, numerical viral load values at VF were described for 17 individuals across 5/11 studies: 2 had VL between 50 and 199 c/mL, 3 had VL between 200 and 1000 c/mL and 7 had VL between 1000 and 10 000 c/mL and 5 had VL above 10 000 c/mL. Among the other 6 studies, VF was categorized as VL ≥50 c/mL (4 individuals), VL ≥200 c/mL (44 individuals) and VL ≥1000 c/mL (2 individuals).

### Resistance associated with mutations at virological failure

Only 4 OCS (*n* = 214 individuals, 15 VF) focused on both VF and resistance outcomes. Genotypic information was provided in 14/15 individuals. The VF rate ranged between 4% and 21.4% (Tables 3 and 4). RAMs emerged in all 14 VF events. The rate of RAM emergence varied between 2.7% and 10.7% for INI and between 0% and 21.4% for NNRTI. Participants with both INI and NNRTI RAMs were reported in three studies: in the ward 86 study, 4/6 individuals with VF developed both NNRTI and INI resistance, 3/6 individuals in D'Amico et al. and one individual in Dawiec et al.

The most prevalent NNRTI RAMs at VF were K101K/E/Q (*n* = 5), Y181Y/C/I (*n* = 5), E138E/K/G (*n* = 4) and M230L (*n* = 3). The most prevalent INI RAMs were Q148R/K (*n* = 5) and E138E/D/K/N (*n* = 3).

### Historic or RNA evidence of baseline resistance in individuals with virological failure

13/14 participants with genotypic information available at VF also had genotypic information available on historical resistance or the presence/absence of RAMs in baseline genotypes (across 4/11 OCS) (Tables 3 and 4). Evidence of historic or baseline resistance was reported in 10/13 participants and the class was specified in 9/10. All baseline RAMs were NNRTI RAMs, and the most prevalent were K103N (*n* = 3), E138G (*n* = 2) and V179I (*n* = 2).

### Management of virological failure

4/14 OCS (*n* = 372) reported post‐VF regimens in 16/36 individuals with VF (Tables 3 and 4). PI‐based regimens were used in 3 cohorts (1–5 individuals/cohort, total *n* = 8), INI + LEN in two individuals, LA‐I CAB + RPV + LEN in one individual, LA‐I CAB + RPV + PI in one individual and PI + LEN in one individual. Three individuals continued on LA‐I CAB + RPV. None of the cohorts reported on whether the PI‐based regimens were taken once or twice daily. Data on re‐suppression was reported in 3 OCS accounting for 10 individuals: 5 re‐suppressed (two on INI + LEN, one on LA‐I CAB + RPV + LEN, two on PIs) and 5 did not (one on LA‐I CAB + RPV + PI and 4 on PIs).

### Discontinuations

Out of the 14 OCS, 10 studies (*n* = 487 individuals) provided information on LA‐I CAB + RPV discontinuation (Tables 2 and 3), with 35 individuals discontinuing in total. Across the 10 OCS, the discontinuation rate ranged from 0% (*n*/*N* = 0/18, 0/31, 0/12, 0/12, 0/10) to 21.4% (*n*/*N* = 6/28). Reasons for discontinuation were erratically reported.

### Case series and OCS including less than 10 individuals with viraemia

We further identified 6 case series (*n* = 7) and 23 OCS (*n* = 76) including fewer than 10 individuals with viraemia (Tables S10 and S11). In most OCS, the baseline characteristics were not available, as the information provided was not stratified between individuals with or without viraemia. Among those, 8 VF occurred in 7 OCS. RAMs emerged in 3/5 VF with corresponding genotypic information. Case studies only reported the successful use of LA‐I CAB + RPV; therefore, no VF was observed.

### Risk of bias

Using a modified Downs and Black bias assessment tool, all 14 OCS were identified as moderate quality or above (scoring more than 50% and showing low risk of bias). The main source of bias was a lack of adjustment for different lengths of follow‐up of patients (11/14 OCS), not reporting all important adverse events (7/14 OCS) and not reporting characteristics of individuals lost to follow‐up, for example, not describing reasons for discontinuation (6/14 OCS) (Table S8).

## DISCUSSION

For people who cannot or will not take oral ART, injectable therapy (where clinically indicated) can be lifesaving. This has led to the DHHS and IAS‐USA providing guidance on the use of LA‐I CAB + RPV in the context of viraemia, advanced HIV and adherence challenges on oral ART.

Substantial evidence exists on the efficacy and safety of LA‐I CAB + RPV in individuals who are virally suppressed [1]. In contrast, the only meta‐analysis that has been performed in individuals with viraemia was small, including just 244 individuals across eight research outputs. It evaluated effectiveness as the sole endpoint due to data heterogeneity, and lacked information on baseline clinical and social characteristics, RAMs and post‐VF regimens, making it difficult to understand the nature and extent of emergent resistance mutations or the implications of VF with resistance in individuals with viraemia at initiation.

This large, granular evidence synthesis adds to the literature by providing a more detailed picture of treatment outcomes in individuals with viraemia in clinical practice.

Most cohorts were situated in North America. We noted that only half the cohorts reported baseline characteristics. When they were reported, 22% (87/400) of participants were assigned female at birth, suggesting that women are under‐represented within the overall cohort relative to the country population. Just under half the OCS reported on ethnicity. In those, racially minoritized individuals represented 68% of the population, although this number might not be representative of all studies, given that those reporting on ethnicity were more likely to intentionally recruit under‐served populations. Social complexity factors such as housing status, mental health and substance use were not commonly described. Importantly, where described, the VF outcomes were not described in the context of socio‐demographic factors, so their effect cannot be determined at an individual level.

In terms of clinical characteristics, median CD4 count was often reported but only 3 cohorts provided data on CD4 threshold <200 cells/mm^3^ and 6 OCS provided information on individuals with very high baseline viral loads >10 000 c/mL. 71% (10/14) of the studies used a single VL above a threshold of 50 or 200 c/mL as VF definition. It is noteworthy that these are highly conservative interpretations of viraemia and do not conform to a recent Delphi consensus on the definitions of VF in the context of LA‐I CAB + RPV. The consensus definitions were: VF: ‘(a) viral load ≥200 copies/mL on two occasions 2‐4 weeks apart, or (b) a single viral load >1000 copies/mL and/or (c) emergent resistance, in the context of timely injections and prior suppression <200 copies/mL, OR (d) unable to suppress viral load to <200 copies/mL on continuous therapy’ [46]. This poses conceptual challenges for evidence synthesis and interpretation, and therefore VF rate should only be interpreted as descriptive, not inferential. Across the 14 cohorts, VF occurred in only 36 individuals of 561 at risk. The VF rate ranged from 0% to 25%, although these numbers should be interpreted cautiously given the small sample size of most studies (8/14 OCS included between 10 and 20 individuals, 4/14 OCS included between 20 and 40 individuals, and 2/14 OCS included more than 100 individuals). In the two OCS which included more than 100 individuals, the VF rate was 4%. The focus of the OCS differed and most (10/14) focused solely on VF and did not provide any information on genotypic resistance. Only four OCS (*n* = 214 individuals, *n* = 15 VF) reported genotypic data on emergent resistance in 14 individuals, all of whom had resistance and 8 of whom had 2 class resistance. Post‐VF regimens were described for 16 individuals. Most received PIs (without specification of whether it was taken once or twice daily) and four received LEN as an anchor agent. Re‐suppression outcomes were unclear.

Notably, as described in the virally suppressed real‐world evidence synthesis, some remained on LA‐I CAB + RPV post VF, which may suggest that although clinicians defined a single elevated VL as a VF in the cohort write‐up, in reality, this did not influence their clinical decision‐making. This emphasizes the need for the scientific community to adopt a consensus definition on VF such as CONSENSUS LA‐I in the context of LA‐I CAB + RPV and subsequent LA‐I regimens [46].

Given the data heterogeneity, this review has several significant limitations. One key limitation is the limited duration of follow‐up of 1 year or less in 11/14 cohorts, which affects the generalizability of the findings. Limited and heterogeneously presented information on key clinical socio‐demographic and clinical baseline characteristics (e.g., CD4 and VL) limits generalizability as well. As stated, the restrictive categorizations of both viraemia and VF affected the review. The absence of genotypic information in 7 OCS with reports of VF (*n* = 22/36 VF) is a significant limitation that may bias interpretation. There is no real‐world evidence from resource‐deprived settings reflecting the lack of global access to this WHO‐recommended therapy. Additionally, few cohorts reported on emergent resistance, prior resistance or on re‐suppression post VF. The evidence synthesis is also limited by publication bias, most especially affecting the case studies, which only reported successful LA‐I CAB + RPV initiations that may lead to over‐confidence in the regimen. Equally, some studies reported socio‐demographic characteristics only in people with VF, which precludes a clear understanding of this relationship. While preliminary evidence is promising, rigorous randomized studies with clear definitions of both viraemia and VF are urgently needed, and the CROWN Study (NCT06694805) on people with viraemia is currently underway. In the interim, adherence to the internationally agreed CONSENSUS‐LAI definitions of VF in randomized trials, observational cohorts and case series would make the interpretation of these studies more rigorous, transparent and generalizable. Conferences and journals could insist on more standardized and stringent reporting of key clinical and socio‐demographic variables, including viraemia and VF definition and a minimum follow‐up duration. These steps would greatly improve the evidence base in this key population.

## AUTHOR CONTRIBUTIONS

C.M.O. and M.S. conceptualized the study and acquired the study funding. The methodology was determined by C.M.O., A.E. and M.S. Elizabeth Ward and Claire Snowball from Inizio Medical performed searches according to the protocol. Ying Jean, Jamie Shorrock and Christopher Dee from Inizio Medical performed the primary screening steps. A.E. and C.M.O. were responsible for secondary study screening and extraction. A.E. and C.M.O. were responsible for quality assessment. A.E., C.P. and C.M.O. were responsible for data analysis. Study findings were interpreted by C.M.O. and C.P. Visualization of the data was conducted by A.E., C.P., M.S. and C.M.O. The original draft of the manuscript was written by C.P. and C.M.O. Reviews and edits of subsequent drafts were conducted by all co‐authors.

## FUNDING INFORMATION

This work was supported by Viiv Healthcare.

## CONFLICT OF INTEREST STATEMENT

Chloe Orkin has received honoraria for advisory boards, lectureships and travel sponsorships from Janssen, Gilead Sciences, ViiV Healthcare, MSD and Bavarian Nordic and has received research grants from Janssen, Gilead Sciences, ViiV Healthcare, MSD and AstraZeneca. Alexa Elias, Chloé Pasin, Melanie Smuk and Amy Paterson have no interests to declare.

## Supporting information


**Data S1.** Supporting information.

## Data Availability

The data that support the findings of this study are available from the corresponding author upon reasonable request.
