# Supplementary material for: Outcomes of real‐world initiation of long‐acting injectable cabotegravir and rilpivirine (LA‐I CAB + RPV) in individuals with viraemia: A systematic review of baseline characteristics, virological failure outcomes and discontinuations
Source: HIV Med. 2025 Sep 13;27(1):42–57. doi: 10.1111/hiv.70113 (PMC12757890; doi:10.1111/hiv.70113)
Supplement: Supplementary file 1 — Data S1. Supporting information. [file HIV-27-42-s001.docx]

**Appendix**

**This appendix is supplement to:**

**Outcomes of real-world initiation of long-acting injectable cabotegravir and rilpivirine (LA-I CAB+RPV) in individuals with viraemia: a systematic review of baseline characteristics, virological failure outcomes and discontinuations.**

Supplement Version Date: 01/9/2025

Contents

[Supplemental Methods 3](#_Toc207616901)

[Search Strategy and Protocol 3](#_Toc207616902)

[Table S1. Search Strategy used via Embase 3](#_Toc207616903)

[Table S2. Search Strategy used via *Pubmed* 4](#_Toc207616904)

[Table S3. Search Strategy used via *Cochrane* 4](#_Toc207616905)

[Table S4. List of 23 HIV-related congresses from which abstracts, posters, and oral presentations were hand-searched 5](#_Toc207616906)

[Inclusion Criteria 5](#_Toc207616907)

[Table S5. PICOS Framework 6](#_Toc207616908)

[Data Extraction 6](#_Toc207616909)

[Table S6. Data extraction fields 6](#_Toc207616910)

[Quality Assessment 7](#_Toc207616911)

[Table S7. Modified Downs and Black Checklist 8](#_Toc207616912)

[Supplemental Results 9](#_Toc207616913)

[Table S8. Risk of bias assessment using modified Downs and Black checklist 9](#_Toc207616914)

[Table S9. Summary data of all 36 VF cases in observational cohort studies 11](#_Toc207616915)

[Table S10. Baseline characteristics of individuals with viraemia in cases series and observational cohort studies including <10 individuals at switch 13](#_Toc207616916)

[Table S11. Data on VF, resistance, post-VF regimens and re-suppression in individuals with viraemia initiating LA-I CAB+RPV in cases series and observational cohort studies including <10 individuals with viraemia at switch 16](#_Toc207616917)

[Appendix References 20](#_Toc207616918)

# Supplemental Methods

## Search Strategy and Protocol

This systematic review followed the Preferred Reporting Items for Systematic Reviews and Meta-Analysis (PRISMA) guidelines. The protocol for the OUTCOMES Study was approved on June 10^th^, 2024. (https://doi.org/10.17605/OSF.IO/V68UN).

A systematic review to identify all observational cohort studies and case series reporting virological outcomes in people with HIV who switched to LA-I CAB+RPV was conducted. The search was first performed on July 21st, 2024, then updated a first time on November 28^th^, 2024 and finally updated on March 17th, 2025. It covered published and grey literature released between January 1st, 2020, and March 17th, 2025. A supplementary search strategy was developed and adapted for use in Embase, Pubmed, and Cochrane (Supplementary Tables S1-S3) and 23 HIV-related congresses were hand searched (Supplementary Table S4). Authors were contacted for clarification of results.

### Table S1. Search Strategy used via Embase

| **No.** | **Query** | **Subject** |
| --- | --- | --- |
| 1 | 'Human immunodeficiency virus'/exp OR 'human immunodeficiency virus infection'/exp | **Disease** |
| 2 | 'human immunodeficiency virus' OR 'human immunedeficiency virus' OR (human AND immun* AND (virus* OR infection*)) |  |
| 3 | 'hiv' OR 'hiv1' OR 'hiv2' OR 'hivi' OR 'hivii' |  |
| 4 | 'acquired immune deficiency syndrome'/exp |  |
| 5 | 'aids' OR 'acquired immunodeficiency syndrome' OR 'acquired immunedeficiency syndrome' OR (acquired AND immun* AND (virus* OR infection* OR syndrome*)) |  |
| 6 | #1 OR #2 OR #3 OR #4 OR #5 |  |
| 7 | 'cabotegravir plus rilpivirine'/exp OR (cabotegravir NEAR/3 rilpivirine) OR (cab NEAR/2 (rpv OR ril)) OR cabenuva OR vocabria OR rekambys | **Intervention** |
| 8 | #6 AND #7 | **Combination** |
|  |  |  |
|  |  |  |
| 9 | #8 NOT (('animal'/exp NOT 'human'/exp) OR ‘In Vitro Study’/exp OR ‘Cell Culture Technique’/exp OR ‘Cell Line’/exp OR ‘ex vivo’:ab,ti OR ‘in vitro’:ab,ti OR ‘ cell line’:ab,ti OR ‘ cell culture’:ab,ti OR ‘animal cell’:ab,ti OR ‘animal tissue’:ab,ti OR ‘animal experiment’:ab,ti OR ‘animal health’:ab,ti OR ‘animal model’:ab,ti OR ‘bird’:ab,ti OR ‘canine’:ab,ti OR ‘dog’:ab,ti OR ‘goat’:ab,ti OR ‘mouse’:ab,ti OR ‘mammal’:ab,ti OR ‘mice’:ab,ti OR ‘murine’:ab,ti OR ‘non human’:ab,ti OR ‘nonhuman’:ab,ti OR ‘rat’:ab,ti OR ‘veterinary’:ab,ti) | **Limited to humans** |
| 10 | #9 NOT ('review':de OR 'editorial':de OR 'addresses':de OR 'biography':de OR 'comment':de OR 'directory':de OR 'festschrift':de OR 'interview':de OR 'legislation':de OR 'news':de OR 'newspaper article':de OR 'patient education handout':de OR 'consensus development conference':de OR 'consensus development conference, nih':de OR 'practice guideline':de OR 'Controlled Clinical Trial':de OR ATLAS:ti OR FLAIR:ti OR 'Phase 1*':ti OR 'Phase I*':ti OR 'Phase 2*':ti OR 'Phase II*':ti OR 'Phase 3*':ti OR 'Phase III*':ti) | **Removal of irrelevant publication types** |
| 11 | #10 AND ('article'/it OR 'article in press'/it OR 'conference abstract'/it OR 'conference paper'/it OR 'conference review'/it OR 'letter'/it) | **Restriction to manuscripts or congress abstracts** |
| 12 | #11 NOT [medline]/lim | **Restriction to Embase-only records** |

### Table S2. Search Strategy used via *Pubmed*

| **No.** | **Query** | **Subject** |
| --- | --- | --- |
| 1 | "HIV"[Mesh] OR "HIV Infections"[Mesh] | **Disease** |
| 2 | "hiv" OR "hiv1" OR "hiv2" OR "hivi" OR "hivii" |  |
| 3 | "human immunodeficiency virus" OR "human immunedeficiency virus" OR ((“human” AND “immun*”) AND ("virus*" OR “infection*”)) |  |
| 4 | "Acquired Immunodeficiency Syndrome"[Mesh] |  |
| 5 | "AIDS" OR "acquired immunodeficiency syndrome" OR "acquired immunedeficiency syndrome" OR ("acquired" AND "immun*" AND ("virus*" OR "infection*" OR "syndrome*")) |  |
| 6 | #1 OR #2 OR #3 OR #4 OR #5 |  |
| 7 | "cabotegravir, rilpivirine drug combination" [Supplementary Concept] OR "rilpivirine cabotegravir"[tiab:~3] OR "CAB RPV"[tiab:~2] OR "CAB RIL"[tiab:~2] OR Cabenuva OR Vocabria OR "Rekambys" | **Intervention** |
| 8 | #6 AND #7 | **Combination** |
| 9 | #8 NOT (("Animals"[MeSH] NOT "Humans"[MeSH]) OR "In Vitro Techniques"[Mesh] OR "Cell Culture Techniques"[Mesh] OR "Cell Line"[Mesh] OR "ex vivo"[tiab] OR "in vitro"[tiab] OR "cell line"[tiab] OR "cell culture"[tiab] OR "animal cell"[tiab] OR "animal tissue"[tiab] OR "animal experiment"[tiab] OR "animal health"[tiab] OR "animal model"[tiab] OR "bird"[tiab] OR "canine"[tiab] OR "dog"[tiab] OR "goat"[tiab] OR "mouse"[tiab] OR "mammal"[tiab] OR "mice"[tiab] OR "murine"[tiab] OR "non human"[tiab] OR "nonhuman"[tiab] OR "rat"[tiab] OR "veterinary"[tiab]) | **Limited to humans** |
|  |  |  |
| 10 |  | **Removal of irrelevant publication types** |
|  | #9 NOT ("Review"[Publication Type] OR "Editorial"[Publication Type] OR "biography"[Publication Type] OR "comment"[Publication Type] OR "directory"[Publication Type] OR "festschrift"[Publication Type] OR "interview"[Publication Type] OR "legislation"[Publication Type] OR "news"[Publication Type] OR "newspaper article"[Publication Type] OR "patient education handout"[Publication Type] OR "consensus development conference"[Publication Type] OR "consensus development conference, nih"[Publication Type] OR "practice guideline"[Publication Type] OR "Controlled Clinical Trial"[Publication Type] OR "ATLAS"[ti] OR "FLAIR"[ti] OR "Phase 1*"[ti] OR "Phase I*"[ti] OR "Phase 2*"[ti] OR "Phase II*"[ti] OR "Phase 3*"[ti] OR "Phase III*"[ti]) |  |
|  |  |  |
|  |  |  |

### Table S3. Search Strategy used via *Cochrane*

| **No.** | **Query** | **Subject** |
| --- | --- | --- |
| 1 | MeSH descriptor: [HIV] explode all trees | **Disease** |
| 2 | MeSH descriptor: [HIV Infections] explode all trees |  |
| 3 | hiv or "hiv-1" or "hiv 1" or "hiv-2" or "hiv 2" or hiv1 or hiv2 or "hiv-i" or "hiv-ii" or "hiv infection" or "hiv-infection" or ("hiv NEXT infect*") or (hiv and infect*) |  |
| 4 | MeSH descriptor: [Acquired Immunodeficiency Syndrome] explode all trees |  |
| 5 | aids or "acquired immunodeficiency syndrome" or "acquired immunedeficiency syndrome" or ("acquired and immun*" and (virus* or infection* OR syndrome*)) |  |
| 6 | #1 or #2 or #3 or #4 or #5 |  |
| 7 | (cabotegravir NEAR/3 rilpivirine) OR (CAB NEAR/2 RPV) OR (CAB NEAR/2 RIL) OR 'Cabenuva' OR Vocabria OR Rekambys | **Intervention** |
| 8 | (#6 AND #7) | **Combination** |

### Table S4. List of 23 HIV-related congresses from which abstracts, posters, and oral presentations were hand-searched

| 1 | Afravih |
| --- | --- |
| 2 | Asian Conference on Hepatitis and AIDS (ACHA) |
| 3 | Australasian HIV & AIDS Conference (ASHM) |
| 4 | National Conference of AIDS Society of India (ASICON) |
| 5 | British Association for Sexual Health and HIV (BASHH) |
| 6 | British HIV Association (BHIVA) |
| 7 | Canadian Conference on HIV/AIDS Research (CAHR) |
| 8 | Conference on Retroviruses and Opportunistic Infections (CROI) |
| 9 | European AIDS Clinical Society (EACS) |
| 10 | European Meeting on HIV and Hepatitis |
| 11 | Grupo de Estudio del SIDA-SEIMC (GeSIDA) |
| 12 | HIV Glasgow |
| 13 | HIV & Hepatitis in the Americas (HIV/HEP) |
| 14 | IDWeek |
| 15 | International AIDS Society/International AIDS Conference (IAS/IAC) |
| 16 | Italian Conference on AIDS and Antiviral Research (ICAR) |
| 17 | International Conference on AIDS and STIs in Africa (ICASA) |
| 18 | International Congress on Infectious Diseases (ICID) |
| 19 | International Workshop on HIV Drug Resistance and Treatment Strategies |
| 20 | The Professional Society for Health Economics and Outcomes Research (ISPOR) |
| 21 | Japanese Society for AIDS Research (JSAR) |
| 22 | Kenya Association of Physicians (KAP) |
| 23 | Société Française De Lutte Contre Le Sida (SFLS) |

## Inclusion Criteria

Multiple stages of screening were conducted. Preliminary screening of reports relevant to the OUTCOMES Study was carried out by two independent reviewers (discrepancies resolved by a third reviewer). Search results were uploaded into Covidence Systematic Review Software, de-duplicated, and then screened using title and abstracts then full text (where applicable). During preliminary screening, outputs which did not describe observational cohort studies (e.g., literature reviews, studies with experimental designs), or which did not evaluate LA-I CAB+RPV treatment for HIV-1 were excluded.

Secondary screening was performed by members of the research team (A.E., with consultation by C.O.) in accordance with a predefined set of inclusion criteria organised using the PICOS framework specifically tailored for Phase 2 of the OUTCOMES Study (Supplementary Table S5). During the screening conducted by the research team, studies and case series were excluded if they did not provide data on virological outcomes (e.g., viral load or clinical endpoints at follow-up). Additionally, mixed cohort studies that reported on both virally suppressed individuals and individuals with viraemia without stratifying the outcomes by viral suppression status were excluded. In those reporting stratified outcomes in both virally suppressed individuals and individuals with viraemia, we only extracted the data for the individuals with viraemia. As per the protocol we designated OCS studies as n>10. Data from OCS with fewer than 10 individuals with viraemia and case series were summarized in a separate table.

When a study yielded multiple research outputs, we prioritised the one with the most recent data. Manuscripts were given preference over congress materials (where available and up to date). Efforts were made to minimise overlap, where feasible. In the event of substantial overlap between multiple outputs, the output with the larger sample was selected.

Inclusion and exclusion criteria for Phase 2 of the OUTCOMES Study were developed based on the following items, organised using the PICOS (Population, Intervention, Comparator, Outcome, Study design) Framework.

### Table S5. PICOS Framework

| Population: | Adults and adolescents living with HIV-1 with viraemia |
| --- | --- |
| Intervention: | Long-acting injectable cabotegravir and rilpivirine |
| Comparator: | Any or none |
| Outcomes: | Virological outcomes at follow-up (e.g., reporting of presence or absence of virological failure, viral suppression (or non-suppression), or other viral load endpoint) |
| Study Design: | Observational cohort studies (e.g., prospective or retrospective observational cohort studies, case reports, analysis of hospital records) including > 10 individuals with viraemia at switch. |

## Data Extraction

Following initial extraction using Microsoft Excel against the broader OUTCOMES Study, the study team (A.E. and cross-checked by C.O.) performed additional extraction for studies that met the inclusion criteria for this review. Information on study characteristics, VF, RAMs, post-VF regimen, and discontinuations in the population with viraemia was extracted and stored in Microsoft Excel based on the following criteria:

### Table S6. Data extraction fields

| Study Characteristics | VF | RAM data | Post-VF data | Discontinuation |
| --- | --- | --- | --- | --- |
| - Study name or first author and year - Study countries - Follow-up time (months) - Dose - VF definition - Number of individuals with viraemia at baseline - Number individuals with viraemia at risk of VF | - Number of VF events, *n (%)* - VL at VF | - Genotype available for baseline and/or historic INI/NNRTI RAMs for those with VF,   *n/N;*  *VF#) RAMs*   - Genotype available at VF,   *n/N*   - VFs with available genotypes with No RAMs,   *n (n/N%, n/VF%)*   - VFs with available genotypes with INI RAMs,   *n (n/N%, n/VF%);*  *VF#) RAMS*   - VFs with available genotypes with NNRTI RAMs,   *n (n/N%, n/VF%);*  *VF#) RAMS*   - VFs with available genotypes with NNRTI & INI RAMs,   *n (n/N%, n/VF%)* | - Post-VF regimen available *(n/N)* - Post-VF regimen,   *drug class;*  *VF#) regimen*   - Re-suppression *n/N, (%);*   *VF#) Y/N/UA* | - Data on discontinuation available (Yes/No) - Number of discontinuations n (%) |

## Quality Assessment

The risk of bias of included studies was assessed using criteria from the Downs and Black Checklist.^1^ Out of 27 items present in the original Downs and Black Checklist,^1^ 16 were excluded in our modified version. We modified the tool in line with previous reviews which also appraised observational cohort studies and studies without an experimental study design.^2,3^ Excluded items concerned randomisation and blinding, measures of effect and statistical tests, and study arms, as this review focused on outcomes observed through descriptive statistics in real-world patients electing to receive LA-I CAB+RPV. Each question received a score of 0 or 1. The modified tool is available in Supplementary Table S7. Similarly to former reviews using the modified checklist,^2,4^ studies fulfilling at least 50% of items were considered moderate or above scientific quality. The assessment was completed by A.E. and cross-checked by M.D. Disagreements in scoring were resolved by a third author (C.O.).

### Table S7. Modified Downs and Black Checklist

| **Section I: Reporting** *(Scoring per question: Yes=1, No=0)* |
| --- |
| 1. Is the hypothesis/ aim/ objective of the study clearly described?  2. Are the main outcomes to be measured clearly described in the Introduction or Methods section?  3. Are the characteristics of the patients included in the study clearly described ?  4. Are the interventions of interest clearly described?  5. Are the main findings of the study clearly described?  6. Have all important adverse events that may be a consequence of the intervention been reported?  7. Have the characteristics of patients lost to follow-up been described? |
| **Section II: External Validity** *(Scoring per question: Yes=1, No=0, Unable to determine=0)* |
| 8. Were the staff, places, and facilities where the patients were treated, representative of the treatment the majority of patients receive? |
| **Section III: Internal Validity- Bias** *(Scoring per question: Yes=1, No=0, Unable to determine=0)* |
| 9. In trials and cohort studies, do the analyses adjust for different lengths of follow-up of patients, or in case-control studies, is the time period between the intervention and outcome the same for cases and controls ?  10. Were the main outcome measures used accurate (valid and reliable)? |
| **Section IV: Internal validity- confounding (selection bias)** *(Scoring per question: Yes=1, No=0, Unable to determine=0)* |
| 11. Were losses of patients to follow-up taken into account? |

| Supplemental ResultsTable S8. Risk of bias assessment using modified Downs and Black checklist | | | | | | | | | | | | | | | |
| --- | --- | --- | --- | --- | --- | --- | --- | --- | --- | --- | --- | --- | --- | --- | --- |
|  | Study | 1. Is the hypothesis/ aim/ objective of the study clearly described? | 2. Are the main outcomes to be measured clearly described in the Introduction or Methods section? | 3. Are the characteristics of the patients included in the study clearly described? | 4. Are the interventions of interest clearly described? | | 5. Are the main findings of the study clearly described? | 6. Have all important adverse events that may be a consequence of the intervention been reported? | 7. Have the characteristics of patients lost to follow-up been described? | 8. Were the staff, places, and facilities where the patients were treated, representative of the treatment the majority of patients receive? | 9. Do the analyses adjust for different lengths of follow-up of patients? | 10. Were the main outcome measures used accurate (valid and reliable)? | 11. Were losses of patients to follow-up taken into account? | **Total, n (%)** | **Grade** |
| **1** | Schneider et al., 2024 (BEYOND) | 1 | 1 | 1 | 1 | | 1 | 1 | 1 | 1 | 1 | 1 | 1 | 11 (100) | Moderate or above |
| **2** | Hessamfar et al., 2024 | 1 | 1 | 1 | 1 | | 1 | 1 | 1 | 1 | 1 | 1 | 1 | 11 (100) | Moderate or above |
| **3** | Dawiec et al., 2024 | 0 | 1 | 1 | 1 | | 0 | 1 | 1 | 1 | 1 | 0 | 1 | 8 (73) | Moderate or above |
| **4** | Jongen et al., 2025 (Dutch ATHENA) | 1 | 1 | 1 | 1 | | 1 | 0 | 0 | 1 | 0 | 1 | 1 | 8 (73) | Moderate or above |
| **5** | Fessler et al., 2024 | 0 | 1 | 0 | 1 | | 1 | 0 | 0 | 1 | 0 | 1 | 1 | 6 (55) | Moderate or above |
| **6** | Christopoulos et al., 2025 (Ward 86) | 0 | 1 | 1 | 0 | | 1 | 1 | 1 | 1 | 0 | 1 | 1 | 8 (73) | Moderate or above |
| **7** | Elion et al., 2023 (Trio) | 1 | 1 | 1 | 1 | | 1 | 0 | 0 | 1 | 0 | 1 | 1 | 8 (73) | Moderate or above |
| **8** | D'Amico et al., 2023 (Compassionate Use) | 1 | 1 | 1 | 1 | | 1 | 1 | 1 | 1 | 0 | 1 | 1 | 10 (91) | Moderate or above |
| **9** | Hsu R, et al., 2023. (OPERA) | 1 | 1 | 1 | 1 | | 1 | 1 | 0 | 1 | 0 | 1 | 1 | 9 (82) | Moderate or above |
| **10** | Brock et al., 2024 | 0 | 0 | 1 | 1 | | 1 | 1 | 1 | 1 | 0 | 1 | 1 | 8 (73) | Moderate or above |
| **11** | Hill et al., 2025 | 1 | 1 | 1 | 1 | | 1 | 0 | 0 | 1 | 0 | 1 | 1 | 8 (73) | Moderate or above |
| **12** | Rousseau et al., 2024 | 1 | 1 | 1 | 1 | | 1 | 0 | 1 | 1 | 0 | 1 | 1 | 9 (82) | Moderate or above |
| **13** | O'Connor, K et al., 2025 | 1 | 1 | 1 | 0 | | 1 | 0 | 0 | 1 | 0 | 1 | 1 | 7 (64) | Moderate or above |
| **14** | Gerber et al., 2025 | 1 | 1 | 1 | 0 | | 1 | 0 | 1 | 1 | 0 | 1 | 1 | 8 (73) | Moderate or above |
| **Total** | | 10/14 | 13/14 | 13/14 | | 11/14 | 13/14 | 7/14 | 8/14 | 14/14 | 3/14 | 13/14 | 14/14 |  |  |
| References: (1) Schneider S, et al. AIDS 2024. Poster THPEB099 (2) Hessamfar M, et al. V Glasgow 2024. Poster P078 (3) Dawiec et al., HIV Glasgow 2025. Poster 097 (4) Jongen et al. Lancet HIV 2025; 12: e40–50 (5) Fessler D, et al. CROI 2024. Poster 1235 (6) Christopoulos et al., CROI 2025. Poster 0683 (7) Elion et al., 2023 ID Week P.1592 (8) D'Amico R, et al. HIV Med 2023;24:202–11 (9) Hsu R, et al. IDWeek 2023. Oral 1028 (10) Brock JB, et al. Clin Infect Dis 2024;78:122–4 (11) Hill et al. J Acquir Immune Defic Syndr 2025;98:185–192 (12) Rousseau A, et al. J Pediatric Infect Dis Soc 2024;13:285–87 (13) O’Connor et al., CROI 2025. Poster 691 (14) Gerber et al., CROI 2025. Poster 682 | | | | | | | | | | | | | | | |

### Table S9. Summary data of all 36 VF cases in observational cohort studies

| **Study characteristics** | | **VF** | | | | **RAM data** | | | | **Post-VF data** | | | |
| --- | --- | --- | --- | --- | --- | --- | --- | --- | --- | --- | --- | --- | --- |
| Study | Total people with viraemia at switch to CAB+RPV, n | VF definition | VF, n (n/N%) | VL at VF (c/mL) | VL at switch to CAB + RPV (c/mL) | Genotype available at VF, n | VF with NNRTI RAM, n (n/N%) | VF with INI RAM, n (n/N%) | Genotypic information available on baseline and/or historic RAM for those with genotypic information at VF, n | VF events with post-VF regimen data | Post-VF regimen type | VF events with known re-suppression outcome | Re-suppressed |
| 1. Schneider et al., 2024 (BEYOND) | 18 | VL >50 c/mL* | 1 (5.6) | VL $\geq$50 (no numerical value available) | VL $\geq$50 (no numerical value available) | NR | NR | NR | NR | NR | NR | NR | NR |
| 2. Hessamfar et al., 2024 | 12 | VL >50 c/mL at 6 months | 3 (25.0) | VL $\geq$50 (no numerical value available) | VL $\geq$50 (no numerical value available) | NR | NR | NR | NR | NR | NR | NR | NR |
| 3. Dawiec et al., 2024 | 18 | NR | 2 (11.1) | VL >1,000  (no numerical value available) | NR | 1/2 | 1 (5.6)  *mutations NR | 1 (5.6)  *mutations NR | 1/1 *mutations NR | NR | NR | NR | NR |
| 4. Jongen et al., 2025 (Dutch ATHENA) | 18 | VF: VL ≥1000 c/mL + ART change or RAMs  CVF: >2 VL> 200 c/ mL or 1 VL >200 c/mL with  CAB or RPV RAMs | 1 (5.6) | 1) VL=200 | 1) VL=100 | 1/1 | 0 (0.0) | 1 (5.6)  N155H | 1/1  None | 1 | 1: PI | 1 | 1: no |
| 5. Fessler et al., 2024 | 31 | VL>50 c/mL | 2 (6.5) | 1) VL=60  2) VL=60 | VL $\geq$50 (no numerical value available) | NR | NR | NR | NR | NR | NR | NR | NR |
| 6. Christopoulos et al., 2025 (Ward 86)* | 150 | VL > 200 c/mL | 6 (4.0) | 1) VL=4,400  2) VL=29,000  3) VL=4,500  4) VL=1300  5) VL=137000  6) VL=8600 | 1) VL=137,000  2) VL=215,000  3) VL=363,800  4) VL=700  5) VL=309  6) VL=540,000 | 6/6 | 6 (4.0)  1) E138K  2) L100I, Y181I  3) K101E  4) K101K/E, K103N, Y181Y/C  5) M230L  6) M230L, K101Q | 4 (2.7)  1) R263K  2) none  3) Q148R  4) E138E/K, Q148K  5) none  6) E138E/D/K/N, G140G/S, S147S/G, Q148K | 5/6  1) T97A (INI)  2) V179I, N348I (minor NNRTI)  3) n/a  4) K103N  5) none  6) V90I | 6 | 1) INI + LEN  2) INI + LEN  3) CAB/RPV + LEN  4) PI  5) PI  6) PI + LEN | 4 | 1) yes  2) yes  3) yes  4) yes  5) NA  6) NA |
| 7. Elion et al., 2023 (Trio) | 24 | VL > 200 c/mL | 3 (12.5) | 1) VL=205  2) VL=1148  3) VL=3715 | 1) VL 50-199 (no numerical value)  2) VL=2,138  3) VL=616 | NR | NR | NR | NR | NR | NR | NR | NR |
| 8. D'Amico et al., 2023 (Compassionate Use) | 28 | VL>50 c/mL | 6 (21.4) | 1) NR  2) VL=799  3) VL=37,594  4) VL=7,190  5) VL=186,972  6) VL=66,000 | 1) VL=61,600  2) VL=32,000  3) VL=116,311  4) VL=77,578  5) VL=205,000  6) VL=1,639,794 | 6/6 | 6 (21.4)  1) E138E/K  2) E138G, M230L  3) E138G, Y181I  4) K101E  5) K101E, Y181Y/C  6) Y181C | 3 (10.7)  1) none  2) Q148R, N155H  3) M50M/I, E157E/Q  4) E138E/K, Q148R  5) none  6) none | 6/6  1) E138G  2) K238K/R, E138G  3) V179I  4) None  5) K103N  6) K103N | 6 | 1) CAB/RPV + PI  2) PI  3) PI  4) PI  5) PI  6) PI | 5 | 1) no  2) no  3) NA  4) no  5) no  6) yes |
| 9. Hsu R, et al., 2023 (OPERA) | 176 | 2 VL ≥200 c/mL  or 1 VL ≥200 + discontinuation (ART change or 2 missed injections) | 7 (4.0) | VL $\geq$200 (no numerical value available) | VL $\geq$50 (no numerical value available) | NR | NR | NR | NR | 3 | CAB+RPV n=3 | NR | NR |
| 11. Hill et al., 2025 | 35 | 2 VL > 200 c/mL | 1 (2.9) | No numerical value available | No numerical value available | NR | NR | NR | NR | NR | NR | NR | NR |
| 14. Gerber et al., 2025 | 17 | VL > 200 c/mL | 4 (23.5) | VL $\geq$200 (no numerical value available) | VL $\geq$200 (no numerical value available) | NR | NR | NR | NR | NR | NR | NR | NR |

### Table S10. Baseline characteristics of individuals with viraemia in cases series and observational cohort studies including <10 individuals at switch

| Study | Individuals with viraemiaat baseline, N | Sex/ Gender* , *M/F* | Transgender women,  *n (n/N%)* | Racially minoritised, *n (n/N%)* | Age, *years* | Alcohol misuse,  *n (n/N%)* | Drug misuse,  *n (n/N%)* | Experiencing homelessness or unstable housing,  *n (n/N%)* | Experiencing mental health issues,  *n (n/N%)* | Years from HIV diagnosis^&^ | Number of prior regimens reported | Drug classes of prior regimens,  *Drug class (Regimen)* |  |
| --- | --- | --- | --- | --- | --- | --- | --- | --- | --- | --- | --- | --- | --- |
| Case Studies | | | | | | | | | | | | | |
| 1. Van Bremen et al., 2023 | 2 | NR | NR | NR | NR | NR | NR | NR | NR | 1. 17 ^‡^ 2. 4 | NR | NR |  |
| 1. Ciccullo et al., 2024 | 1 | F | NR | NR | 49 | NR | NR | NR | NR | 25 | 2 | PI (FTC/TDF+DRV/r)  INI (TAF/FTC+DTG) |  |
| 1. Cunningham-Smith et al., 2024 | 1 | F | 0 | 0 | 44 | NR | 1 (100) | 1 (100) | NR | NR | 2 | INI (BIC/FTC/TAF)  NNRTI (DOR/3TC/TDF) |  |
| 1. Jørgensen and Larsen, 2023 | 1 | F | NR | NR | 42 | NR | NR | NR | NR | 7 | 3 | NNRTI (EFV/FTC/TDF)  NNRTI (RPV/FTC/TDF)  PI (DRV/FTC/TDF) |  |
| 1. Al-Handola, et al., 2023 | 1 | M | NR | NR | 47 | NR | 1 (100) | NR | NR | 27 | 1 | Multi-core agent (ETR + DTG + DRV/r) |  |
| 1. Barnett et al., 2022 | 1 | F | NR | NR | 53 | NR | NR | NR | NR | 16 | 6 | NNRTI (EFV/TDF/FTC)  INI (DTG/ABC/3TC)  PI (DRV/COBI+TAF/FTC)  INI (DTG+TAF/FTC)  INI (BIC/TAF/FTC)  INI (DTG-3TC) |  |
| Mixed cohorts including <10 individuals with viraemia at switch ^£^ | | | | | | | | | | | | | |
| 1. Bissuel et al., 2024 | 9^@^ | NR | NR | NR | NR | NR | NR | NR | NR | NR | 1. 3 | NNRTI (EFV/FTC/TDF)  INI (BIC/FTC/TAF)  PI (TDF/FTC + DRV/r) |  |
|  |  |  |  |  |  |  |  |  |  |  | 2-9) NR | NR |  |
| 1. Taramasso et al., 2024 | 7 | NR | NR | NR | NR | NR | NR | NR | NR | NR | NR | NR |  |
| 1. O’Connor, LF et al., 2025 | 6 | NR | NR | NR | NR | NR | NR | NR | NR | NR | NR | NR |  |
| 1. González-Cordón et al., 2024 | 5^@^ | 1. M | NR | NR | 50 | NR | NR | NR | NR | NR | NR | INI (ABC/3TC/DTG) |  |
|  |  | 2) M |  |  | 41 |  |  |  |  |  |  | Multicore agent (DTG/RPV) |  |
|  |  | 3-5) NR |  |  | NR |  |  |  |  |  |  | NR |  |
| 1. Iannone et al., 2025 (a) (Unconventional Use) | 5 | NR | NR | NR | NR | NR | NR | NR | NR | NR | NR | NR |  |
| 1. Koutsoupias et al., 2024 | 4 | NR | NR | NR | NR | NR | NR | NR | NR | NR | NR | NR |  |
| 1. Bowden et al., 2024 | 4 | NR | NR | NR | NR | NR | NR | NR | NR | NR | NR | NR |  |
| 1. Fernández-Hinojal et al., 2023 | 4 | 1. F | NR | NR | 57 | NR | NR | NR | NR | 23 | NR | INI (TAF/FTC+DTG) |  |
|  |  | 2) F |  |  | 58 |  |  |  |  | 11 |  | INI (DTG/3TC) |  |
|  |  | 3) M |  |  | 59 |  |  |  |  | 9 |  | INI (DTG/3TC) |  |
|  |  | 4) M |  |  | 60 |  |  |  |  | 34 |  | Multicore agent (DTG/RPV) |  |
| 1. Perez et al., 2024 | 4 | NR | 0 | NR | NR | NR | 4 | NR | NR | NR | NR | NR |  |
| 1. Ogilvy et al., 2024 | 4 | NR | NR | NR | NR | NR | NR | NR | NR | NR | NR | NR |  |
| 1. Nasser et al., 2023 | 3 | NR | NR | NR | NR | NR | NR | NR | NR | NR | NR | NR |  |
| 1. Seang et al., 2023 | 3 | 1. M | NR | NR | 36 | NR | NR | NR | NR | NR | NR | NR |  |
|  |  | 2) M |  |  | 54 |  |  |  |  |  |  |  |  |
|  |  | 3) M |  |  | 49 |  |  |  |  |  |  |  |  |
| 1. Andrea et al., 2025 | 3 | NR | NR | NR | >65 | NR | NR | NR | NR | NR | NR | NR |  |
| 1. Rossotti et al., 2022 | 2 | NR | NR | NR | NR | NR | NR | NR | NR | NR | NR | NR |  |
| 1. Zingaropoli et al., 2025 | 2 | NR | NR | NR | NR | NR | NR | NR | NR | NR | NR | NR |  |
| 1. Serris et al., 2024 | 2 | NR | NR | NR | NR | NR | NR | NR | NR | NR | NR | NR |  |
| 1. Haser et al. 2024 | 2 | NR | NR | NR | NR | NR | NR | NR | NR | NR | NR | NR |  |
| 1. Dannenberg et al., 2024 | 2 | NR | NR | NR | NR | NR | NR | NR | NR | NR | NR | NR |  |
| 1. Williams et al., 2025 | 1 | M | 0 | NR | NR^¥^ | NR | NR | NR | NR | NR | NR | NR |  |
| 1. Lázaro-Martín et al., 2024 | 1 | F | NR | NR | 32 | NR | NR | NR | NR | NR | NR | NR |  |
| 1. Acosta et al., 2024 | 1 | NR | NR | NR | NR | NR | NR | NR | NR | NR | NR | NR |  |
| 1. Torralba et al., 2024 | 1 | NR | NR | NR | NR | NR | NR | NR | NR | NR | NR | NR |  |
| 1. Iannone et al., 2025 (b) | 1 | NR | NR | NR | NR | NR | NR | NR | NR | NR | NR | NR |  |
| F: Female, M: Male, NR: Not reported, INI: Integrase Inhibitor, NNRTI: Non-Nucleoside Reverse Transcriptase Inhibitor, PI: Protease Inhibitor  *We considered female individuals as those reported in observational cohort studies as any of the following: female gender or sex, cis-gender women; ^&^ Where the year (date) of diagnosis was reported, we calculated the number of years from diagnosis by subtracting the year of diagnosis from the year they initiated LAI CAB+RPV; ‡ Deduced from the information provided that the individual initiated oral ART in 2004 and switched to CAB+RPV in 2021; ^£^ If relevant data were provided by the study output but it did not discretely describe data for individuals with viraemia, we considered this NR; @ Baseline characteristics were only reported in individuals with VF; ¥ Cohort included adolescents ages 12-24  References: **(1)** Van Bremen K, et al. HIV Med 2023;24 Suppl 1:30–31 **(2)** Ciccullo A, et al. AIDS Res Hum Retrovirus 2024. doi: 10.1089/aid.2023.0057 **(3)** Cunningham-Smith L, et al. BASHH 2024. Abstract P0050 **(4)** Jørgensen NM and Larsen L. EACS 2023. Poster EP.CC.040 **(5)** Al-Handola, et al. Eur J Case Rep Intern Med 2023;10:003981 **(6)** Barnett SK, et al. AIDS 2022;36:1475–6 **(7)** Bissuel et al., SFLS 2024. Poster VIEI18 **(8)** Taramasso et al., HIV Glasgow 2024 Poster 066 **(9)** O’Connor LF, et al. AIDS Res Hum Retroviruses. 2025 Apr;41(4):181-188 **(10)** González-Cordón A et al. HIV Glasgow 2024. Poster P080 **(11)** (a) Iannone et al. BMC Infect Dis 2025;25:105 **(12)** Koutsoupias P, et al. IDWeek 2024. Poster P-573 **(13)** Bowden B, et al. ASHM HIV&AIDS 2024. Poster **(14)** Fernández-Hinojal F, et al. EACS 2023. Poster eP.A.026 **(15)** Perez A, et al. Open Forum Infect Dis 2024;11(11):ofae640 **(16)** Ogilvy A, et al. IDWeek 2024. Poster P-572 **(17)** Nasser K, et al. EACS 2023. Poster eP.T2.03 **(18)** Seang S, et al. EACS 2023. Poster 783 **(19)** Andrea et al., CROI 2025. Poster 677 **(20)** Rossotti R, et al. ICAR 2022. Poster OP63 **(21)** Zingaropoli et al., CROI 2025 Poster 372 **(22)** Serris A, et al. J Antimicrob Chemother 2024;79:2932–38 **(23)** Haser GC, et al. AIDS Res Hum Retroviruses 2024 **(24)** Dannenberg C, et al. HIV Glasgow 2024. Poster P111 **(25)** Williams et al., Pediatr Infect Dis J 2025 Jan 24;44(7):650-656 **(26)** Lázaro-Martín et al., GeSIDA 2024. Poster 101 **(27)** Acosta et al., GeSIDA 2024. Poster 027 **(28)** Torralba et al., GeSIDA 2024. Poster 028 **(29)** (b) Iannone et al., AIDS Behav 2025;29:1228–34 | | | | | | | | | | | | |  |

### Table S11. Data on VF, resistance, post-VF regimens and re-suppression in individuals with viraemia initiating LA-I CAB+RPV in cases series and observational cohort studies including <10 individuals with viraemia at switch

| 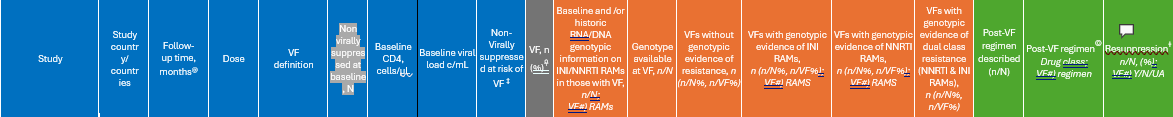**Study characteristics** | | | | | | | | **VF** | **RAM data^&^** | | | | | | **Post-VF data** | | | |
| --- | --- | --- | --- | --- | --- | --- | --- | --- | --- | --- | --- | --- | --- | --- | --- | --- | --- | --- |
| Study | Country | VF definition | Individuals with viraemia at risk of VF ^‡^ | Dose | Baseline viral load c/mL | Baseline CD4, cells/μL | Follow-up time, months^@^ | VF, n (%)^☥^ | Baseline and /or historic RNA/DNA genotypic information on INI/NNRTI RAMs in those with VF,  *n/N; RAMs* | Genotype available at VF, *n/N* | VFs without genotypic evidence of resistance, *n (n/N%, n/VF%)* | VFs with genotypic evidence of INI RAMs,  *n (n/N%, n/VF%); RAMS* | VFs with genotypic evidence of NNRTI RAMs,  *n (n/N%, n/VF%); RAMS* | VFs with genotypic evidence of dual class resistance (NNRTI & INI RAMs),  *n (n/N%, n/VF%)* | Post-VF regimen described (n/N) | Post-VF regimen^©^  *Drug class (Regimen)* | Resuppression^ⵜ^ *n/N, (%)* |  |
| Case Studies | | | | | | | | | | | | | | | | | | |
| 1. Van Bremen et al., 2023 | Germany | VL>40 c/mL | 2 | Q2M | 1. 15,762 c/mL | 101 cells/μL | 2* | 0 (0) |  |  |  |  |  |  |  |  |  |  |
|  |  |  |  |  | 1. 350,239 c/mL | 4 cells/μL | 1.6 |  |  |  |  |  |  |  |  |  |  |  |
| 1. Cicculo et al., 2024 | Italy | VL>50 c/mL | 1 | Q1M | 1. 392 c/mL | 80 cells/μL | 2.8 | 0 (0) |  |  |  |  |  |  |  |  |  |  |
| 1. Cunningham-Smith et al., 2024 | UK | NR | 1 | Q2M | 1. 47,000 c/mL | NR | 2 | 0 (0) |  |  |  |  |  |  |  |  |  |  |
| 1. Jørgensen and Larsen, 2023 | Denmark | VL>50 c/mL | 1 | Q1M | 1. 230,000 c/mL | 240 cells/μL | 1.8 | 0 (0) |  |  |  |  |  |  |  |  |  |  |
| 1. Al-Handola, et al., 2023 | USA | VL>40 c/mL | 1 | Q1M | 1. >300,000 c/ml | <10 cells/μL | 6 | 0 (0) |  |  |  |  |  |  |  |  |  |  |
| 1. Barnett et al., 2022 | USA | VL>20 c/mL | 1 | Q1M | 1. 341,000 c/mL | 260 cells/μL | 6 | 0 (0) |  |  |  |  |  |  |  |  |  |  |
| Mixed cohorts including <10 individuals with viraemia at switch^£^ | | | | | | | | | | | | | | | | | | |
| 1. Bissuel et al., 2024 | France | NR | 9 | Q2M | 1. 13,830 c/mL ^✖^ | NR | 9 | 1 (0.1) | NR | NR | NR | NR | NR | NR | NR | NR | NR |  |
|  |  |  |  |  | 2-9) NR |  |  |  |  |  |  |  |  |  |  |  |  |  |
| 1. Taramasso et al., 2024 | Italy | NR | 7 | Q2M | - 1. NR ^$^ | NR | NR | 0 (0) |  |  |  |  |  |  |  |  |  |  |
| 1. O’Connor LF et al., 2025 | USA | 2 VL >1,000c/mL | 6 | NR | 1-2) >50 c/mL | NR | NR | 0 (0) |  |  |  |  |  |  |  |  |  |  |
| 1. González-Cordón et al., 2024 | Spain | 2 VL>50 c/mL | 5 | Q2M | 1. 154 c/mL | NR | 6.4 | 2 (0.4) | NR | 2/2 | 0 (0, 0) | 74I, 163R | 138K, 230I | 2 (0.4, 100) | 2/2 | 2 PI (DRVc/FTC/TAF) | NR |  |
|  |  |  |  |  | 1. 88 c/mL |  |  |  |  |  |  | 140S | 138K, 230I |  |  |  |  |  |
|  |  |  |  |  | 3-5) >50 c/mL |  |  |  |  |  |  |  |  |  |  |  |  |  |
| 1. Iannone et al., 2025 (a) (Unconventional Use) | Italy | 2 VL > 50 c/  ml or 1 VL > 200 c/ml | 5 | Q2M | 1-5) >200 c/mL | NR | 12 | 0 (0) |  |  |  |  |  |  |  |  |  |  |
| 1. Koutsoupias et al., 2024 | USA | VL>201 c/mL | 4 | NR | 1. 294,000 c/mL | NR | NR | 1 (0.3) | NR | 1/1^¥^ | 0 (0,0) | NR | NR | NR | NR | NR | NR |  |
|  |  |  |  |  | 1. 7,200 c/mL |  |  |  |  |  |  |  |  |  |  |  |  |  |
|  |  |  |  |  | 1. 1,210 c/mL |  |  |  |  |  |  |  |  |  |  |  |  |  |
|  |  |  |  |  | 1. 90 c/mL |  |  |  |  |  |  |  |  |  |  |  |  |  |
| 1. Bowden et al., 2024 | Australia | VL ≥200 c/mL | 4 | Q2M | 1. 790,000 c/mL | NR | 8.2 | 0 (0) |  |  |  |  |  |  |  |  |  |  |
|  |  |  |  |  | 1. 90,000 c/mL |  | 5.5 |  |  |  |  |  |  |  |  |  |  |  |
|  |  |  |  |  | 1. 183 c/mL |  | NR |  |  |  |  |  |  |  |  |  |  |  |
|  |  |  |  |  | 1. 80 c/mL |  | 13.8 |  |  |  |  |  |  |  |  |  |  |  |
| 1. Fernández-Hinojal et al., 2023 | Spain | VL ≥50 c/mL | 4 | Q2M | 1. 50 c/mL | NR | 1.5 | 1 (0.3) | NR | NR | NR | NR | NR | NR | NR | NR | NR |  |
|  |  |  |  |  | 1. 838 c/mL |  |  |  |  |  |  |  |  |  |  |  |  |  |
|  |  |  |  |  | 1. 85 c/mL |  |  |  |  |  |  |  |  |  |  |  |  |  |
|  |  |  |  |  | 1. 61 c/mL |  |  |  |  |  |  |  |  |  |  |  |  |  |
| 1. Perez et al., 2024 | USA | VL ≥200 c/ml | 4 | Q2M | 1-2) 201–1000 c/mL | ≥200 cells/μL | NR | 0 (0) |  |  |  |  |  |  |  |  |  |  |
|  |  |  |  |  | 3-4) 1001–5000 c/mL |  |  |  |  |  |  |  |  |  |  |  |  |  |
| 16. Ogilvy et al., 2024 | USA | VL>200 c/mL | 4^ | NR | 1. 50 c/mL | 496 | 5 | 1 (0.3) | NR | 1/1 | 1 (0.3,100) | 0 (0, 0) | 0 (0, 0) | 0 (0, 0) | NR | NR | 1/1 (100) |  |
|  |  |  |  |  | 1. 310 c/mL | 1144 | 17 |  |  |  |  |  |  |  |  |  |  |  |
|  |  |  |  |  | 1. 90 c/mL | 966 | 12 |  |  |  |  |  |  |  |  |  |  |  |
|  |  |  |  |  | 1. 51-200 c/mL | NR^ | NR^ |  |  |  |  |  |  |  |  |  |  |  |
| 1. Nasser et al., 2023 | USA | VL>200 c/mL | 3 | NR | 1-3) >200 c/mL | NR | 11 | 0 (0) |  |  |  |  |  |  |  |  |  |  |
| 1. Seang et al., 2023^%^ | France | 2 VL> 200 c/mL | 3^%^ | Q2M | 1. 260 c/mL | NR | 2 | 1 (0.3) | 1/1; None | 1/1 | 1 (0.3 ,100) | 0 (0, 0) | 0 (0, 0) | 0 (0, 0) | 1/1 | NNRTI (TDF/FTC/DOR) | 1/1 (100) |  |
|  |  |  |  |  | 2) 250 c/mL |  | 3 |  |  |  |  |  |  |  |  |  |  |  |
|  |  |  |  |  | 3) 69 c/mL |  | 2 |  |  |  |  |  |  |  |  |  |  |  |
| 1. Andrea et al., 2025 (GEPPO Cohort) | Italy | VL ≥50 c/mL | 3 | Q2M | 1. 945 c/mL | NR | NR | 0 (0) |  |  |  |  |  |  |  |  |  |  |
|  |  |  |  |  | 2) 234 c/mL |  |  |  |  |  |  |  |  |  |  |  |  |  |
|  |  |  |  |  | 3) 55 c/mL |  |  |  |  |  |  |  |  |  |  |  |  |  |
| 1. Rossotti et al., 2022 | Italy | NR | 2 | NR | 1-2) NR | NR | NR | 0 (0) |  |  |  |  |  |  |  |  |  |  |
| 1. Zingaropoli et al., 2025 | Italy | NR | 2 | Q2M | 1-2) >50 c/mL | NR | 12 | 0 (0) |  |  |  |  |  |  |  |  |  |  |
| 1. Serris et al., 2024 | France | VL>200 c/mL+ discontinuation OR 2 VL>50 c/mL | 2 | Q2M | 1. 91 c/mL | NR | NR | 0 (0) |  |  |  |  |  |  |  |  |  |  |
|  |  |  |  |  | 1. 55 c/mL |  |  |  |  |  |  |  |  |  |  |  |  |  |
| 1. Haser et al. 2024 | USA | VL>200 c/mL | 2 | Q1M | 1. 767 c/mL | NR | 21 | 0 (0) |  |  |  |  |  |  |  |  |  |  |
|  |  |  |  | Q1M & Q2M | 1. 50-200 c/mL | NR | >25 |  |  |  |  |  |  |  |  |  |  |  |
| 1. Dannenberg et al., 2024 | Germany | VL>200 c/ml | 2 | Q2M | 1. >200 c/mL | NR | 15 | 1 (0.5) | NR | NR | NR | NR | NR | NR | NR | NR | NR |  |
|  |  |  |  |  | 1. 50-200 c/mL |  | 12 |  |  |  |  |  |  |  |  |  |  |  |
| 1. Williams et al., 2025 | USA | VL>20 c/mL | 1 | NR | 1. 90 c/mL | NR | NR | 0 (0) |  |  |  |  |  |  |  |  |  |  |
| 1. Lázaro-Martín et al., 2024 | Spain | NR | 1 | Q2M | 1. 38,000 c/ml | 143 cells/µL | 10.8 | 0 (0) |  |  |  |  |  |  |  |  |  |  |
| 1. Acosta et al., 2024 | Spain | NR | 1 | Q2M | 1. 64 c/ml | NR | 3 | 0 (0) |  |  |  |  |  |  |  |  |  |  |
| 1. Torralba et al., 2024 | Spain | NR ^#^ | 1 | Q2M | 1. 90 c/mL | NR | 13 | 0 (0) |  |  |  |  |  |  |  |  |  |  |
| 1. Iannone et al., 2025 (b) | Italy | 2 VL ≥ 50 c/mL or ≥ 1 VL≥ 1,000 c/mL | 1 | Q2M | 1. 86 c/mL | NR | 5.5 | 0 (0) |  |  |  |  |  |  |  |  |  |  |
| NR: Not reported, Q1M: monthly, Q2M: 2-monthly, VL: Viral load, VF: Viral failure, INI: Integrase Inhibitor, NNRTI: Non-Nucleoside Reverse Transcriptase Inhibitor, PI: Protease Inhibitor, RAMs: resistance-associated mutations, RNA: Ribonucleic acid, DNA: Deoxyribonucleic acid  ^&^RAMs not listed under INI or NNRTI in the Stanford Algorithm were excluded. n/N RAMs rate was calculated using the total number individuals with viraemia at risk of VF as the denominator; ^‡^ individuals with viraemia included in the analysis;^@^Where months were not provided, we calculated months using the average number of weeks (4.35) or days (30.44) per month, or the number of injections received at follow-up; ^☥^ VF rates were calculated using the total number of individuals with viraemia at risk of VF as the denominator; ^©^When multiple post-VF regimens were listed, we reported the one provided at the moment of re-suppression (if data available), or otherwise the first regimen listed; ^ⵜ^Based on definition of suppression or individuals reported as ‘resuppressed’ by individual studies; ^£^ If relevant data were provided by the study output but it did not discretely describe data for individuals with viraemia, we considered this NR; * Estimated based on information that LAI CAB+RPV was initiated in July 2021 and follow-up occurred September 2021; ^✖^ This data was only provided for those who experienced VF; ^$^ Individuals were described as ‘non-suppressed’ at baseline; ^¥^ Emergence of RAMs reported, but RAMs class NR; ^Baseline CD4 and follow-up time only reported for those with viraemia at follow-up (n=3); ^%^ Data were only provided for those who discontinued LAI CAB+RPV; ^#^VF definition involves a confirmed viral load measurement (‘CVF’)  References: **(1)** Van Bremen K, et al. HIV Med 2023;24 Suppl 1:30–31 **(2)** Ciccullo A, et al. AIDS Res Hum Retrovirus 2024. doi: 10.1089/aid.2023.0057 **(3)** Cunningham-Smith L, et al. BASHH 2024. Abstract P0050 **(4)** Jørgensen NM and Larsen L. EACS 2023. Poster EP.CC.040 **(5)** Al-Handola, et al. Eur J Case Rep Intern Med 2023;10:003981 **(6)** Barnett SK, et al. AIDS 2022;36:1475–6 **(7)** Bissuel et al., SFLS 2024. Poster VIEI18 **(8)** Taramasso et al., HIV Glasgow 2024 Poster 066 **(9)** O’Connor LF, et al. AIDS Res Hum Retroviruses. 2025 Apr;41(4):181-188 **(10)** González-Cordón A et al. HIV Glasgow 2024. Poster P080 **(11)** (a) Iannone et al. BMC Infect Dis 2025;25:105 **(12)** Koutsoupias P, et al. IDWeek 2024. Poster P-573 **(13)** Bowden B, et al. ASHM HIV&AIDS 2024. Poster **(14)** Fernández-Hinojal F, et al. EACS 2023. Poster eP.A.026 **(15)** Perez A, et al. Open Forum Infect Dis 2024;11(11):ofae640 **(16)** Ogilvy A, et al. IDWeek 2024. Poster P-572 **(17)** Nasser K, et al. EACS 2023. Poster eP.T2.03 **(18)** Seang S, et al. EACS 2023. Poster 783 **(19)** Andrea et al., CROI 2025. Poster 677 **(20)** Rossotti R, et al. ICAR 2022. Poster OP63 **(21)** Zingaropoli et al., CROI 2025 Poster 372 **(22)** Serris A, et al. J Antimicrob Chemother 2024;79:2932–38 **(23)** Haser GC, et al. AIDS Res Hum Retroviruses 2024 **(24)** Dannenberg C, et al. HIV Glasgow 2024. Poster P111 **(25)** Williams et al., Pediatr Infect Dis J 2025 Jan 24;44(7):650-656 **(26)** Lázaro-Martín et al., GeSIDA 2024. Poster 101 **(27)** Acosta et al., GeSIDA 2024. Poster 027 **(28)** Torralba et al., GeSIDA 2024. Poster 028 **(29)** (b) Iannone et al., AIDS Behav 2025;29:1228–34 | | | | | | | | | | | | | | | | | |  |

# Appendix References

1. Downs SH, Black N. The feasibility of creating a checklist for the assessment of the methodological quality both of randomised and non-randomised studies of health care interventions. J Epidemiol Community Health. 1998;52(6):377-84.

2. Shivakumar S, Srivastava A, G CS. Body Mass Index and Dental Caries: A Systematic Review. Int J Clin Pediatr Dent. 2018;11(3):228-32.

3. Canuto R, da Silva Garcez A, Kac G, de Lira PIC, Olinto MTA. Eating frequency and weight and body composition: a systematic review of observational studies. Public Health Nutr. 2017;20(12):2079-95.

4. Silva AE, Menezes AM, Demarco FF, Vargas-Ferreira F, Peres MA. Obesity and dental caries: systematic review. Rev Saude Publica. 2013;47(4):799-812.
